# Biofilm synergy by *Agrobacterium deltaense* and *Bacillus velezensis* in co-cultures indicates bacterial interspecific cooperation

**DOI:** 10.1016/j.bioflm.2026.100364

**Published:** 2026-05-04

**Authors:** Yue Zhang, Zhigang Wang, Weihui Xu

**Affiliations:** a College of Life Science and Agroforestry, Qiqihar University, Qiqihar, 161006, China; b Heilongjiang Provincial Engineering Technology Research Center of Agromicrobial Preparation Industrialization, Qiqihar, 161006, China; c Heilongjiang Provincial Collaborative Innovation Center of Agrobiological Preparation Industrialization, Qiqihar, 161006, China; d Heilongjiang Provincial Concept Verification Center of High Concentration Organic Waste Liquid Biotransformation and Industrialization, Qiqihar, 161006, China

**Keywords:** Multi-species biofilms, Consortium, Synergism, Metabolic interactions

## Abstract

Rhizosphere microorganisms typically coexist within complex multispecies biofilm communities. However, the interspecies interactions within these biofilm-associated microbial consortia remain largely unexplored. A synthetic bacterial SynCom Q was previously constructed in our laboratory for controlling watermelon Fusarium wilt. This study simplified SynCom Q into SynCom Y (*Agrobacterium deltaense* LSQ16 and *Bacillus velezensis* WB) using a stepwise strain omission strategy and combined genome-scale modeling with multi-omics analyses to reveal mechanisms of synergistic biofilm formation. Co-cultivation of *A. deltaense* LSQ16 and *B. velezensis* WB significantly enhanced biofilm formation. Compared with monocultures, co-cultivation also markedly increased extracellular polysaccharide production and metabolic activity. SynCom Y effectively enhances resistance to the pathogen through the promotion of biofilm formation. Compared with monocultures, co-cultivation led to the upregulation of genes associated with extracellular polysaccharides (EPS) synthesis, biofilm formation, and quorum sensing pathways. Meanwhile, microbes within the mixed-species biofilm cooperatively regulated the production of the extracellular matrix to enhance biofilm development. Predictive results indicated metabolite exchange between the two strains involved amino acids, purines, phosphates, and other compounds. Moreover, exogenous supplementation of l-ornithine and guanine further enhanced the biofilm-forming capacity of *B. velezensis* WB. Our results indicate that a synergistic biofilm was formed through metabolic cooperation by *Agrobacterium deltaense* LSQ16 and *Bacillus velezensis* WB in co-cultures.

## Highlights

• Strains *Agrobacterium deltaense* LSQ16 and *Bacillus velezensis* WB synergistically interacted through metabolic cooperation.
• Mixed-species biofilms enhance biofilm structural stability and environmental adaptability.
• Co-cultivation promotes the DEGs associated with quorum sensing and the c-di-GMP signaling pathway.

## Graphical abstract

**Figure:**
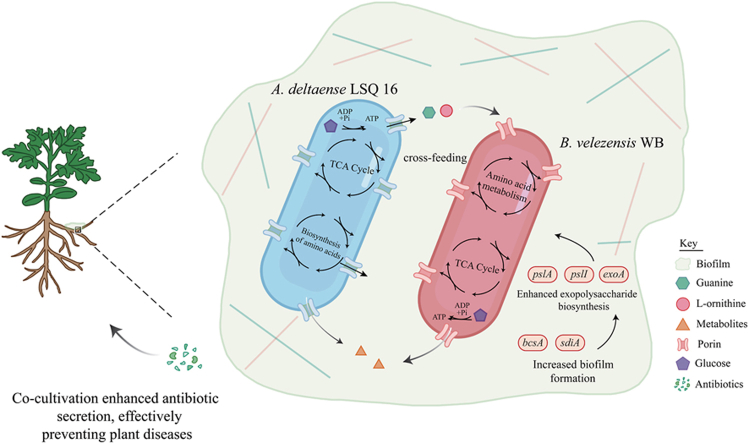

## 1 Introduction

Biofilms are an effective bacterial strategy for coping with environmental stresses [1], promoting plant growth and nutrient uptake, and exhibiting great potential in biological control [2]. Biocontrol bacteria establish biofilms on plant leaves and roots to achieve effective colonization [3], thereby protecting plants from pathogenic microorganisms [4]. Simultaneously, they reduce the availability of nutrient for pathogens and block potential sites for colonization, thereby inhibiting the growth of pathogens [5,6]. Microbes in biofilms usually form complex communities rather than existing as single cells [7]. Microbial communities promote biofilm formation through interspecies interactions [8,9], and influence the distribution and biomass of bacteria within the biofilm matrix. Interspecies interactions can be classified as synergistic, antagonistic, or neutral, with synergistic interactions being more prevalent in multispecies biofilm systems [10]. Compared with single-species biofilms, mixed-species biofilms form highly convoluted structures [11] and exhibit greater thickness [12,13]. Mixed-species biofilms can also enhance stress tolerance [14] by increasing metabolic activity and facilitating horizontal gene transfer among bacteria [15]. This enables better adaptation to environmental changes and plays a crucial role in the biological control of soil-borne plant diseases [[16], [17], [18]].

A dual-species consortium of *Pseudomonas protegens* Δ*pltB* and *Bacillus velezensis* DMW1 synergistically enhanced biofilm formation and provided better control of tomato bacterial wilt than *P. protegens* alone [19]. A consortium of *Pantoea agglomerans* and *Bacillus subtilis* forms biofilms, enhances defense enzyme activities in potato, and exhibits pronounced antagonistic activity against plant pathogens [20]. Compared with single strains, synthetic communities enhance collective function via interspecific synergy, thereby improving disease control efficacy. Fusarium wilt in watermelon, which is caused by the *Fusarium oxysporum* f. sp. *niveum* (Fon), is a severe soil-borne disease and has become one of the major constraints on watermelon production. However, studies on controlling watermelon Fusarium wilt using synthetic microbial consortia from a biofilm perspective, and elucidating interspecies interactions within these consortia, remain lacking. Current studies primarily investigate microbial interactions using traditional approaches, such as cross-feeding of strain metabolites. However, microbial interactions are diverse and complex, and conventional methods are limited in capturing specific metabolic exchanges between strains.

Genome-scale metabolic models (GEMs) can be used to predict metabolic exchanges between strains during microbial community construction and simplification [21]. Previous studies have revealed metabolic interactions between strains using GEMs and omics analyses, with in vitro experiments validating the model predictions [16,22]. Predicted metabolic exchanges between *Bacillus velezensis* SQR9 and *Pseudomonas stutzeri* XL272 suggest that exchanging branched-chain amino acids, like leucine and valine, helps alleviate environmental stress and promotes cucumber growth [16]. GEMs analyzed interspecies interactions in a dual-species consortium of *Acinetobacter* sp. AG3 and *Bacillus* sp. R45, revealing that the exchange of small molecules such as l-serine and hypoxanthine can drive consortium growth [23]. Therefore, integrating GEMs with multi-omics analyses holds great potential for investigating interactions between microbial strains.

This study was based on the SynCom Q, previously constructed in our laboratory for the biocontrol of watermelon Fusarium wilt [24]. Building on this, the present study simplified the consortium via stepwise strain omission and employed modeling and multi-omics approaches to explore the cooperative biofilm-forming mechanisms in the SynCom Y. The two-strain consortium establishes a nutritional relationship through the exchange of metabolites. The pivotal role of these metabolites in facilitating biofilm development was further validated by exogenous supplementation experiments. By elucidating the cooperative biofilm-forming mechanisms within SynCom Y, this study provides critical theoretical insights for the rational design of biofilm-based microbial consortia for disease management.

## 2 Methods and materials

### 2.1 Antagonistic interactions among strains

#### 2.1.1 Solid-air interface biofilm formation

This study was based on SynCom Q [24], developed by the Microbial Ecology Laboratory of Qiqihar University for biocontrol of watermelon Fusarium wilt, consisting of *Enterobacter ludwigii* LSQ1, *Acinetobacter pittii* LSQ3, *Chitinophaga silvisoli* LSQ14, *Agrobacterium deltaense* LSQ16, *Bacillus velezensis* LSQ19, and *Bacillus velezensis* WB (Table S1). Glycerol-preserved strains were inoculated into TSB and cultured overnight at 25 °C with shaking at 150 rpm. Cultures were adjusted to an OD_600_ of 0.8, and 1.5 μL aliquots were spotted onto LBGM solid medium (Table S2), either individually or in pairwise combinations. For pairwise assays, inoculation points were placed 5 cm apart (2.5 cm on either side of the plate center). Plates were sealed with parafilm and incubated at 25 °C for 3 days to evaluate inter-strain antagonistic interactions.

#### 2.1.2 Metabolic facilitation assay

Strains were inoculated into TSB and incubated in the dark at 25 °C with shaking at 150 rpm for 72 h until reaching the decline phase. Cultures were centrifuged at 10,000 rpm for 10 min, and the supernatants were collected and filtered through a 0.22 μm sterile membrane (Millex, PES) to remove residual cells, yielding cell-free supernatants (CFS) for each strain.

The strains used for inoculation were pre-cultured in TSB until they reached the exponential phase and then adjusted to the same optical density (OD_600_ = 1.0). In each well of a 96-well plate, 178 μL of TSB, 20 μL of CFS, and 2 μL of bacterial suspension were added. TSB without CFS served as the control [25]. All treatments were incubated statically at 30 °C for 48 h, with three replicates per treatment. The promotion or inhibition between strains was defined by the OD_600_ ratio of treatment to control (OD_600_ spent/fresh) [25], with >1 indicating promotion and <1 indicating inhibition.

### 2.2 Biofilm formation ability assay

#### 2.2.1 Quantification of biofilm formation by use of crystal violet

Strains were pre-cultured, and their optical density at 600 nm (OD_600_) was adjusted to 0.8. Subsequently, they were inoculated at 10% (*v*/*v*) into 2 mL of LBGM medium. For single-species biofilms, the bacterial suspension was directly introduced into 24-well plates. For mixed-species biofilms suspensions of different strains were mixed in equal volumes before being added to the plates. Samples were incubated statically at 30 °C for 24, 48, and 72 h to compare the biofilm-forming capacities of different strain combinations.

Biofilm formation was quantified using the crystal violet staining method [10]. The culture medium in each well was carefully removed, and the biofilms were rinsed 2–3 times with sterile water to remove non-adherent cells. After air-drying at room temperature, 0.1% crystal violet solution was added and incubated for 20 min. The biofilms were then washed five times with sterile water and air-dried to remove residual moisture. Subsequently, the bound dye was solubilized with an acetone–ethanol solution (80:20, *v*/*v*) for 15 min and thoroughly mixed. Finally, biofilm formation was quantified by measuring the absorbance at 540 nm using a microplate reader. Synergy was defined as occurring when (Abs_540_ MS − SD) > (Abs_540_ BS + SD) [10]. MS and BS represent the absorbance of mixed- and single-species biofilms at 540 nm, respectively; SD denotes the standard deviation. Each treatment includes three replicates.

#### 2.2.2 Biofilm biomass quantification assay

Biofilms were cultivated as described in Section 2.2.1. A six-well plate was used, with a sterile 100 μm pore-size nylon mesh cell strainer (Biologix) placed in each well. Each well was supplemented with 10 mL TSB medium and 100 μL bacterial suspension and incubated statically at 30 °C for 24, 48, and 72 h to allow biofilm formation on the nylon mesh cell strainer. After incubation, the cell strainers were removed and excess surface liquid was gently blotted with filter paper before weighing. The fresh biofilm weight was calculated by subtracting the weight of the sterile nylon mesh. The samples were then dried in an oven for 24 h and weighed to determine the dry biofilm weight. Each treatment was performed in triplicate.

### 2.3 Interaction mechanisms within SynCom Y

#### 2.3.1 Strain-specific qPCR on biofilm and planktonic fractions

Single- and dual-species biofilms were cultured as described in Section 2.2.1. After 24 h of incubation, cells from both the biofilm and the planktonic fractions were collected. The culture medium in each well was carefully removed and transferred to a centrifuge tube to collect the planktonic cells. The biofilms were rinsed 2–3 times with PBS to remove non-adherent cells, after which the biofilm matrix was harvested to collect biofilm-associated cells. All samples were centrifuged at 9000 rpm for 4 min and stored at −80 °C. Quantitative PCR was used to determine the cell numbers of each strain in both biofilm and planktonic fractions [12]. Specific primers were designed based on the 16S rRNA sequences of *A. deltaense* LSQ16 and *B*. *velezensis* WB using Premier 5.0 software (Table S3). Total DNA was extracted using the TIANamp Bacteria DNA Kit, and *q*PCR was performed in a 10 μL reaction containing 5 μL of 2 × SYBR Green Master Mix, 0.2 μL of each primer (10 μM/L), 1 μL of template DNA, and 3.6 μL of ddH_2_O. A 2692 bp pMD18-T plasmid was used for the standard curve (Fig. S1). Gene copy numbers were normalized by strain-specific 16S rRNA gene copy numbers from whole-genome data and converted to cell-equivalent abundance. Each treatment had three replicates.

#### 2.3.2 Chemotaxis assay

Strain chemotaxis toward bacterial metabolites was assessed using a modified assay [26]. *A. deltaense* LSQ16 and *B. velezensis* WB were pre-cultured to the exponential phase in TSB and adjusted to an OD_600_ of 1.0. The CFS of each strain was used as the chemoattractant and was prepared as described in Section 2.1.2. Sterile water and TSB served as negative and positive controls, respectively. A 30 μL bacterial suspension and 30 μL of chemotactic solution were placed 1 cm apart on a sterile slide. An inoculating loop was used to connect the bacterial suspension and chemotactic solution on both sides of the slide. Samples were left in a laminar flow hood for 15 min, after which the chemotactic solution was collected and live cells were counted microscopically. Bacterial concentration was calculated as follows:

Bacterial concentration = Total count / 5 × 4000 × 1000 × dilution factor

A significantly higher number of viable cells compared to the control indicated a chemotactic response toward the metabolite. Each treatment had three replicates.

#### 2.3.3 Analysis of biofilm metabolic activity

Biofilm cell metabolic activity was measured using the XTT reduction assay [12]. Single- and dual-species biofilms were cultured as described in Section 2.2.1. After 24 h of incubation, the biofilms were washed twice with 0.1 M PBS to remove planktonic cells. Each well added 10 μL menadione, 200 μL XTT, and 790 μL PBS, incubated at 37 °C in the dark for 3 h, and OD_492_ was measured to assess metabolic activity. Each treatment had five replicates.

#### 2.3.4 Quantitative analysis of water-insoluble polysaccharides

The content of water-insoluble extracellular polysaccharides was determined using the anthrone–sulfuric acid method [12]. Single- and dual-species biofilms were cultured as described in Section 2.2.1. After the biofilms were washed with 0.1 M PBS, they were harvested and resuspended in 1 mL of 0.1 M PBS. The suspension was centrifuged at 6000 rpm for 10 min to remove the supernatant, and this step was repeated three times. Then, 1 mL of 1 M NaOH was added, and the mixture was stirred for 2 h to extract water-insoluble extracellular polysaccharides. The solution was mixed with anthrone–sulfuric acid (1:3, *v*/*v*), heated at 95 °C for 5 min, and then cooled to room temperature. The absorbance at 625 nm was measured, and the extracellular polysaccharide concentrations in the biofilms were calculated using a standard curve. Each treatment had five replicates.

### 2.4 Effect of watermelon root exudates on the biofilm-forming capacity

Watermelon seedlings at the two-leaf stage were transplanted into a split-root system. In this system, half of the roots growing in non-sterile soil and the other half in sterile distilled water. Containers for split-root were wrapped with aluminum foil to prevent light exposure. When watermelon seedlings developed 4–5 leaves, root exudates were harvested from the side containing sterile distilled water. The exudates were filtered through 0.22 μm filters, collected, and then stored at 4 °C until use.

Biofilm cultivation was carried out as described in Section 2.2.1. Specifically, 25, 50, 100, 200, 300, 400, 500, 600, 700, and 800 μL of root exudates were added to the LBGM medium, respectively. An equal volume of sterile water was used as a blank control. Samples were incubated statically at 30 °C for 24 h. Biofilm formation was quantified using the crystal violet staining method, with five replicates per treatment.

### 2.5 Resistance of multi-species biofilms to fusaric acid

To investigate the effect of fusaric acid (FA, Sigma-Aldrich) on the biofilm-forming ability of the simplified consortium, FA was first dissolved in methanol to prepare a 50 μg/mL stock solution. Subsequently, this stock solution was diluted to final concentrations of 1, 3, 5, 7, 9, 11, and 13 μg/mL. Different concentrations of FA were introduced at 10% (*v*/*v*) into 2 mL of LBGM medium. An equal volume of methanol was added as a control. Single- and dual-species biofilms were cultured as described in Section 2.2.1. Biofilm formation was observed and quantified by crystal violet staining, with three replicates for each treatment.

### 2.6 Pot experiment

Pot experiments were conducted to evaluate the control effects of singles strains and consortium on Fusarium wilt in watermelon. The soil properties were as follows: pH 5.95, electrical conductivity 0.38 ms/cm, organic matter 20.33 g/kg, total N 1.9 g/kg, available K 78.0 mg/kg, and available P 19.3 mg/kg. The experiments contained five groups as follows: *A. deltaense* LSQ16, *B. velezensis* WB, SynCom Y, SynCom Q and CK (sterile TSB). Each group consisted of three replicates, with each replicate containing ten pots (10 cm × 10 cm), and one watermelon seedings was planted in each pot. Watermelon seedlings with two-leaf were treated with bacterial suspensions (1 × 10^8^ CFU/g soil), followed by Fon inoculation (1 × 10^7^ CFU/g soil) three days later, with bacterial suspensions applied every 7 days. Disease incidence was assessed 30 days after Fon inoculation, and the disease index and control efficacy were calculated [27]. The disease index was scored on a 0-4 scale: 0, normal growth; 1, wilting <1/4; 2, wilting affecting 1/4-1/2 of the plant; 3, wilting affecting 1/2-3/4 of the plant; 4, whole-plant wilting or death.

Disease index (%) = [∑ (Number of diseased plants at each grade × Disease grade) / (Total number of plants investigated × 4)] × 100%

Control efficacy (%) = (Disease index of control group – Disease index of treatment group) / Disease index of treatment group × 100%

### 2.7 Metabolomic analysis of single- and mixed-species biofilms

Single- and mixed-species biofilms were cultured as described in Section 2.2.1. After a 24 h incubation, planktonic cells were carefully removed using sterile PBS. Subsequently, the biofilms were incubated in sterile saline at room temperature for 3 h. The mixture underwent ultrasound treatment, followed by centrifugation at 4 °C for 3 min to collect biofilm samples, which were subsequently lyophilized [28]. Six replicates were carried out for each sample.

Dried samples were added to 400 μL of 80% methanol (containing 0.02 mg/mL internal standard) in 2 mL centrifuge tubes for metabolite extraction. The solution was ground at −10 °C for 6 min at 50 Hz, then sonicated for 30 min at 5 °C and 40 kHz. Samples were left at −20 °C for 30 min, followed by centrifugation at 4 °C at 13,000 rpm for 15 min. The supernatant was collected for LC-MS analysis. UHPLC-MS/MS was performed using the UHPLC-Q Exactive system (Thermo Fisher). The raw sequencing data have been uploaded to the MetaboLights database (https://www.ebi.ac.uk/metabolights/MTBLS12444) under the accession number MTBLS12444.

### 2.8 Macrotranscriptomic analysis of single- and mixed-species biofilms

Single- and mixed-species biofilms were cultured as described in Section 2.2.1. After a 24 h incubation, planktonic cells were removed and washed three times with PBS. Biofilm was scraped into 1 mL of PBS, centrifuged at 5000 rpm for 5 min, and then resuspended in 100 μL of PBS. The samples were frozen in liquid nitrogen and stored at −80 °C for RNA extraction and RNA-seq. Five replicates were performed for each sample [29].

Total RNA was extracted using TRIzol® (Invitrogen), followed by treatment with DNase I (TaKaRa) to remove genomic DNA. RNA purity and concentration were measured using the 2100 Bioanalyzer (Agilent) and the NanoDrop 2000 (Thermo). RNA was subjected to sequencing on the Illumina HiSeq X Ten/NovaSeq 6000 (2 × 150 bp). The levels of differentially expressed genes (DEGs) were calculated using Transcripts Per Million (TPM) [30]. Subsequently, differential expression analysis was carried out with DESeq2 (http://bioconductor.org/). Genes with a fold change ≥2 or ≤0.5 and *P-*value <0.05 considered as significant [31]. The raw sequencing data have been deposited in the NCBI database (https://www.ncbi.nlm.nih.gov/) under accession numbers SRR33382738–SRR33382752.

### 2.9 Metabolic model construction and interspecies metabolic exchange analysis

CarveMe was employed to reconstruct draft genome-scale metabolic models for *A. deltaense* LSQ16 and *B. velezensis* WB [32], which were then used to simulate their growth and metabolite production in LBGM medium. The models were refined and validated using the Cobra toolbox [33]. Flux balance analysis (FBA) was performed to assess the metabolic interaction potential of the two-species consortium [34] and identify all potentially exchangeable metabolites.

### 2.10 In vitro validation experiments

Guanine, ornithine, malic acid, and guanosine were added as additional carbon sources during biofilm cultivation of *B. velezensis* WB and SynCom Y, whereas serine, myristic acid, and pyroglutamic acid were individually supplemented as additional carbon sources during biofilm cultivation of *A. deltaense* LSQ16 and SynCom Y. All metabolites were used at a concentration of 0.5 g/L. Cultures were incubated statically at 30 °C for 24 h. Biofilm formation was observed and quantified using the crystal violet staining method.

### 2.11 Statistical analyses

Statistical analysis was performed using SPSS 23 (SPSS, Chicago, IL, USA). Data are presented as mean ± SD from biological replicates. Student's t-test was used for two-group comparisons, and one-way ANOVA for multiple-group comparisons. *P* < 0.05 was considered statistically significant. Data visualization was performed using R 4.0.3 or GraphPad Prism 8.

## 3 Results

### 3.1 Formation ability of mono- and mixed-species biofilms

**Fig. 1:**
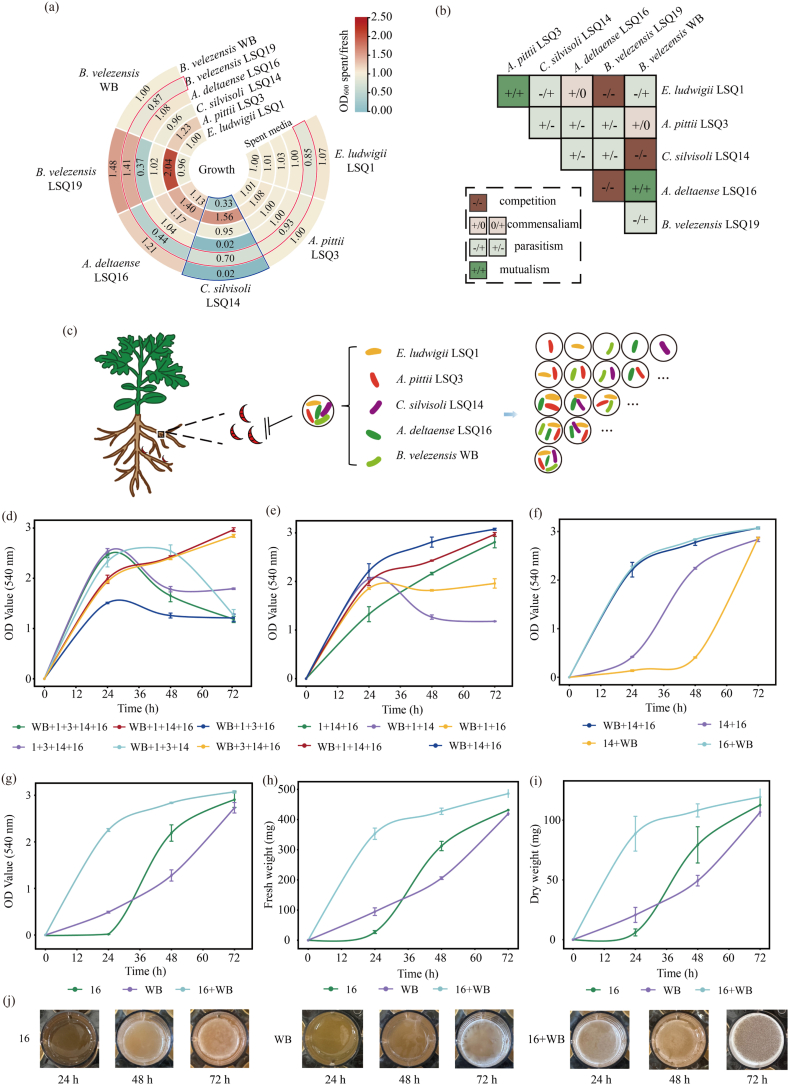
Microbiota simplification. (a) The average OD_600_ of different bacterial strains cultured with cell-free supernatants. (b) A pairwise interaction matrix was generated using the OD_600_ spent/fresh values. (c) Schematic of synthetic community simplification. (d-g) The biofilm formation was measured by crystal violet staining at three time points: 24, 48 and 72 h. (h-i) Fresh and dry biomass of biofilms formed by SynCom Y in monoculture and co-culture at 24 h, 48 h, and 72 h. (j) Biofilm images of *A. deltaense* LSQ16, *B. velezensis* WB, and SynCom Y at 24, 48, and 72 h. Note: In Fig. 1a, blue trapezoids show effects of other strains' metabolites on the target strain, and horizontal rings show effects of the target strain's metabolites on others. OD_600_ spent/fresh >1 indicates promotion, <1 indicates inhibition. Interactions where the ratio was significantly greater than 1 (*P*< 0.05) are indicated with (+), interactions where it was significantly less than 1 are indicated with (−), and interactions with a ratio equal to 1 are represented as (0). (For interpretation of the references to colour in this figure legend, the reader is referred to the Web version of this article.)

This study comprehensively assessed the compatibility among strains within SynCom Q and the impact of strain composition on biofilm formation, enabling a systematic simplification of the consortium. Solid–air interface biofilm assays and cross-feeding experiments were used to investigate antagonistic interactions among the strains. The results showed that *Agrobacterium deltaense* LSQ16 and *Bacillus velezensis* WB, as well as *Enterobacter ludwigii* LSQ1 and *Acinetobacter pittii* LSQ3, exhibited synergistic interactions (Fig. 1a–b). In Fig. 1a, vertical trapezoids show effects of other strains' metabolites on the target strain, and horizontal rings show effects of the target strain's metabolites on others. Red and blue regions indicate the strongest inhibition in horizontal and vertical comparisons, respectively. The blue region indicates that the metabolites of *E. ludwigii* LSQ1, *A. deltaense* LSQ16, and *B. velezensis* WB all inhibited the growth of *Chitinophaga silvisoli* LSQ14. The red region indicates that metabolites of *Bacillus velezensis* LSQ19 broadly inhibited the growth of *E. ludwigii* LSQ1, *A. pittii* LSQ3, *C. silvisoli* LSQ14, *A. deltaense* LSQ16, and *B. velezensis* WB. Compared with the blue region, where only *C. silvisoli* LSQ14 was inhibited, the red region shows that *B. velezensis* LSQ19 exerts a broader inhibitory effect on the entire consortium. In the solid–air interface assay, the colony diameters of *B. velezensis* LSQ19 and *B. velezensis* WB in monoculture were 2.0 cm and 2.1 cm, respectively. In co-culture, the colony diameter of *B. velezensis* WB decreased to 1.8 cm, indicating that *B. velezensis* LSQ19 inhibited the growth of *B. velezensis* WB (Fig. S2). Therefore, LSQ19 was excluded from the consortium, resulting in a five-strain community. Building on this, a stepwise strain omission strategy (Fig. 1c) was used to sequentially remove individual strains, generating different combinations, which were then screened and simplified based on biofilm formation. In the first-round screening, WB+1 + 14+16 and WB+3 + 14+16, which exhibited similar growth, were selected for second-round simplification (Fig. 1d). The WB+14 + 16 combination displayed the highest biofilm formation in both parallel simplification procedures (Fig. 1, Appendix A). Following the third-round screening and comparison with the biofilm formation of individual strains, the combination with the highest biofilm biomass was identified as SynCom Y, consisting of *A. deltaense* LSQ16 and *B. velezensis* WB (Fig. 1g–i). Meanwhile, biofilm biomass of *E. ludwigii* LSQ1 and *A. pittii* LSQ3 was quantified in mono- and co-culture, showing that SynComY exhibited a higher biofilm-forming capacity (Fig. S5). To rule out potential quantitative bias of the crystal violet assay, biofilm biomass was further evaluated by fresh and dry weight measurements across different consortia. The results showed a high consistency between biofilm biomass and the crystal violet assay (Fig. 1h–i, Fig. S6). The co-cultivation of the strains significantly increased biofilm biomass, which steadily increased at 24, 48 and 72 h. The biofilm accumulation rate was high between 24 and 48 h, and maximum biomass was reached at 72 h. Therefore, SynCom Y was chosen for further experiments.

### 3.2 *A. deltaense* LSQ16 facilitates the growth of B. velezensis WB

**Fig. 2:**
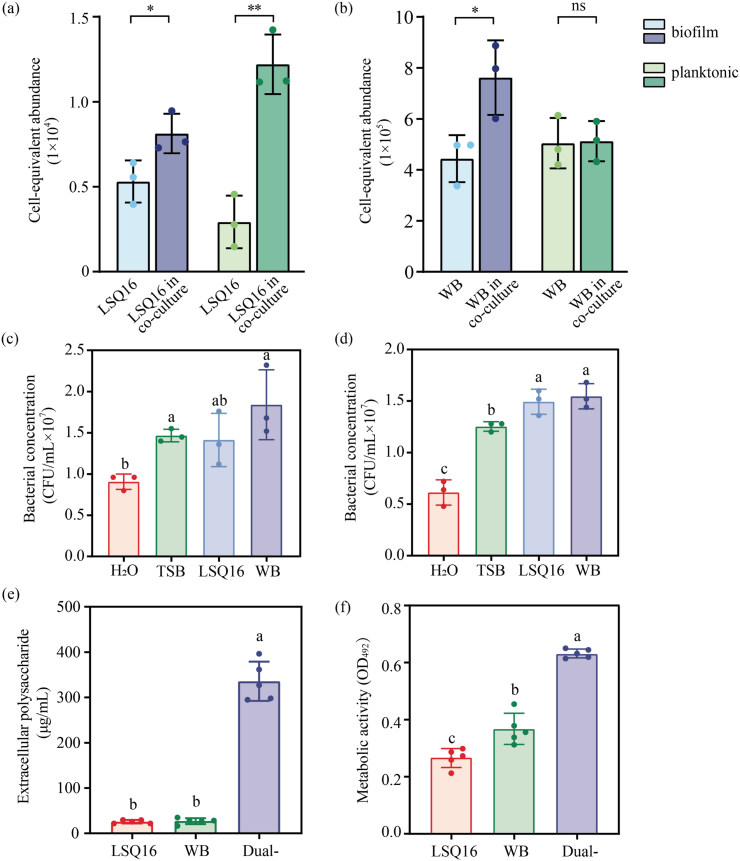
Investigation of interactions between strains within consortium Y. (a-b) Quantitative detection of *A. deltaense* LSQ16 and *B. velezensis* WB in planktonic and dual-species biofilms states under mono- and co-culture conditions. Significance was determined by a paired *t*-test. Statistical significance is indicated by asterisks. ∗*P* < 0.05, “ns" indicates no significant difference. (c) Chemotaxis of *A. deltaense* LSQ16 toward different chemoattractants. Statistical testing was performed using a one-way ANOVA with Tukey's multiple comparisons test (*P* < 0.05). Data represents the mean ± SD of n = 3 biological replicates. (d) Chemotaxis of *B*. *velezensis* WB toward different chemoattractants. Statistical testing was performed using a one-way ANOVA with Tukey's multiple comparisons test (*P* < 0.05). Data represents the mean ± SD of n = 3 biological replicates. (e-f) Determination of extracellular polysaccharide content and metabolic activity in single- and dual-species biofilms. Statistical testing was performed using a one-way ANOVA with Tukey's multiple comparisons test (*P* < 0.05). Data represents the mean ± SD of n = 5 biological replicates.

When co-cultured with *B. velezensis* WB, the cell abundance of *A. deltaense* LSQ16 increased in both biofilm and planktonic fractions (Fig. 2a). For *B. velezensis* WB, co-cultivation did not have a significant impact on its growth in the planktonic fraction but markedly enhanced its growth in biofilms (Fig. 2b). In chemotaxis assays, *A. deltaense* LSQ16 showed no chemotactic response to its own metabolites or those of *B. velezensis* WB (Fig. 2c), whereas *B. velezensis* WB exhibited chemotaxis toward metabolites of both *A. deltaense* LSQ16 and itself (Fig. 2d).

Water-insoluble extracellular polysaccharides (EPS) are the major components of the biofilm matrix. The cellular metabolic activity and EPS content of single- and mixed-species biofilms were measured, with the EPS standard curve shown in Fig. S7. Results demonstrated that EPS levels were relatively low in monocultures of *A. deltaense* LSQ16 and *B. velezensis* WB, reaching 25.64 ± 3.86 μg/mL and 26.96 ± 6.66 μg/mL, respectively. In co-culture, EPS levels increased significantly, reaching 335.65 ± 43.33 μg/mL (Fig. 2e), representing a 12.45–13.09-fold increase compared with the monocultures. This indicates that co-cultivation markedly promoted EPS synthesis. In single-species biofilms, the cellular metabolic activity of *B. velezensis* WB was higher than that of *A. deltaense* LSQ16, with values of 0.38 ± 0.06 and 0.27 ± 0.03, respectively. The cellular metabolic activity in dual-species biofilms was 0.65 ± 0.02, indicating a 1.72- to 2.38-fold increase, which was significantly higher than that in single-species biofilms (Fig. 2f).

### 3.3 The inter-strain interactions stabilized cooperation

**Fig. 3:**
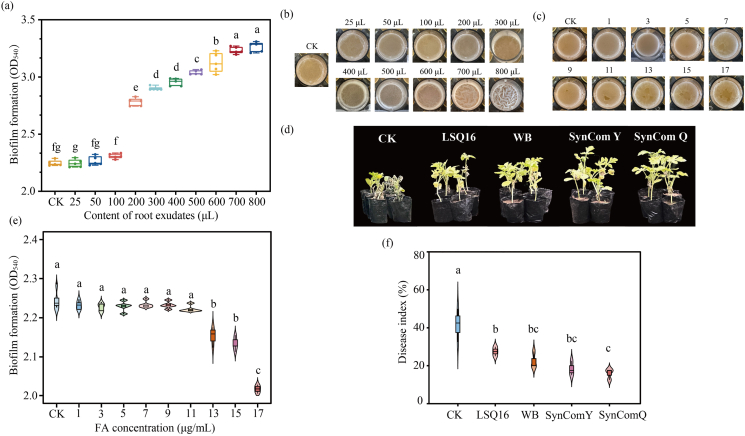
Effect of simplified SynComs on Fusarium wilt in watermelon. (a) The biofilm formation was assessed by crystal violet staining after the addition of different contents of root exudates. Statistical testing was performed using a one-way ANOVA with Tukey's multiple comparisons test. Data represents the mean ± SD of n = 5 biological replicates (*P* < 0.05). (b) Biofilm images under different concentrations of root exudates. (c) Biofilm images under different FA concentrations. (d) Pot experiment. (e) The biofilm formation was measured by crystal violet staining after the addition of different concentrations of fusaric acid (FA). Statistical testing was performed using a one-way ANOVA with Tukey's multiple comparisons test. Data represents the mean ± SD of n = 3 biological replicates (*P* < 0.05). (f) Disease index of watermelon Fusarium wilt under different treatments. (For interpretation of the references to colour in this figure legend, the reader is referred to the Web version of this article.)

Different contents of root exudates were added at the onset of mixed-species biofilm cultivation to assess whether root exudates influence biofilm formation. The results showed that when root exudates were added in low volumes (25-100 μL), biofilm formation was weak. As the amount of exudates increased, the mixed-species biofilm displayed progressively enhanced biofilm-forming capacity (Fig. 3a–b). At 600 μL of root exudates, the mixed-species biofilm developed a wrinkled architecture, markedly enhancing biofilm formation in SynCom Y.

Fusaric acid (FA) inhibited biofilm formation in a concentration-dependent manner, with stronger inhibition observed at higher concentrations. Biofilm biomass declined for *A. deltaense* LSQ16 at 7 μg/mL FA and for *B. velezensis* WB at 13 μg/mL (Fig. S8). The two-strain biofilm showed a notable biomass decrease at 17 μg/mL FA, indicating enhanced resistance to FA compared with single-species biofilms (Fig. 3c and e).

Pot experiments were conducted to assess the disease-suppressing abilty of SynCom Y. At 30 dpi (Fon), watermelon seedlings exhibited symptoms of Fusarium wilt. The SynCom Y, composed of *A. deltaense* LSQ16 and *B. velezensis* WB, exhibited a disease incidence of 18.33% and a control efficacy of 79.13%, which was significantly higher than that of the single-strain treatments (Fig. 3d and f, Table S4).

### 3.4 Untargeted metabolomic analysis of biofilm formation

**Fig. 4:**
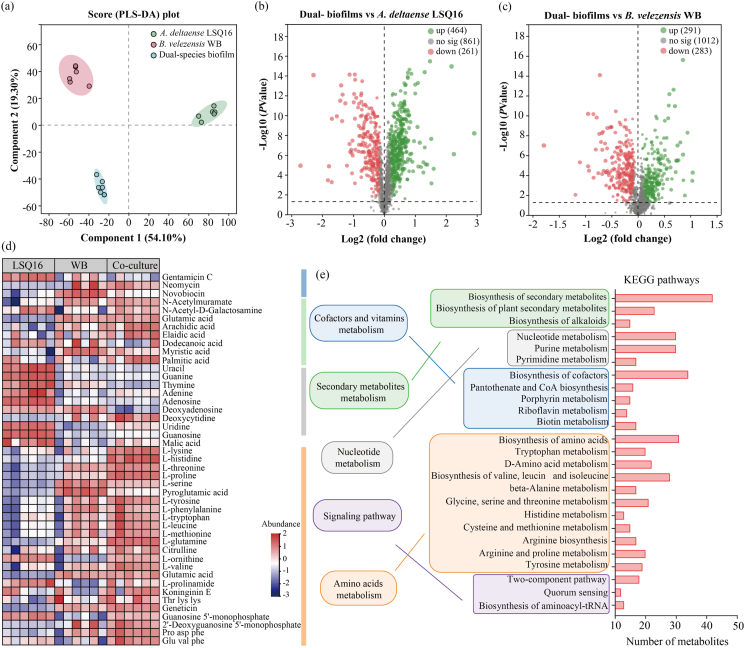
Analysis of differentially expressed metabolites in co-culture and monoculture conditions. (a) PLS-DA analysis of single- and dual-species biofilms. (b) Volcano plot of differentially expressed metabolites in *A. deltaense* LSQ16 when co-cultured with *B. velezensis* WB compared with *A. deltaense* LSQ16 monoculture. (c) Volcano plot of differentially expressed metabolites in *B. velezensis* WB when co-cultured with *A. deltaense* LSQ16 compared with *B. velezensis* WB monoculture. (d) Overview of differential metabolites under monoculture and co-culture conditions for the two strains. (e) KEGG pathway analysis of differential metabolites under co-culture and monoculture conditions for the two strains.

Metabolomic analysis was performed on *A. deltaense* LSQ16 and *B. velezensis* WB in both monoculture and co-culture. Supervised partial least squares discriminant analysis (PLS-DA) showed a clear metabolic separation between co-culture and monoculture (Fig. 4a). Compared with *A. deltaense* LSQ16, 464 metabolites were upregulated in the co-culture (Fig. 4b); compared with *B. velezensis* WB, 291 metabolites were upregulated in the two-species biofilm (Fig. 4c).

Differential metabolites were mainly enriched in pathways related to cofactor and vitamin metabolism, secondary metabolites metabolism, and nucleotide metabolism. Amino acid-related pathways were also significantly enriched, including histidine metabolism, tryptophan metabolism, d-amino acid metabolism, tyrosine metabolism, and the biosynthesis of valine, leucine, and isoleucine (Fig. 4e). Further analysis of amino acid-related compounds revealed that essential amino acids, including l-histidine, l-threonine, l-tryptophan, l-valine, and l-leucine, were significantly more abundant in the co-culture. Non-essential amino acids, including l-proline and l-tyrosine, were also significantly enriched in the co-culture, indicating that co-cultivation promotes amino acid biosynthesis (Fig. 4d). Biosynthetic pathways of secondary metabolites were significantly enriched, with antibiotics such as neomycin markedly increased in the co-culture (Fig. 4d), indicating that co-cultivation of the two strains synergistically activates antibiotic biosynthesis and secondary metabolite pathways (Fig. 4e). Pyrimidine metabolism pathways were significantly enriched in co-culture, with compounds such as deoxycytidine markedly increased. Meanwhile, guanosine-5′-monophosphate and 2′-deoxyguanosine-5′-monophosphate were significantly increased in co-culture compared with monocultures of *B. velezensis* WB and *A. deltaense* LSQ16, respectively, suggesting that purine nucleotide metabolism may be activated to support the energy (ATP/GTP) and nucleic acid synthesis required for biofilm formation.

Pyrimidine metabolism was significantly enriched in the co-culture, with compounds such as deoxycytidine, guanosine-5′-monophosphate, and 2′-deoxyguanosine-5′-monophosphate markedly increased, indicating active pyrimidine metabolism. Nucleotide derivatives (5′-deoxyguanosine monophosphate) indicate activated nucleotide biosynthesis, supporting energy (ATP/GTP) and nucleic acid demands during co-culture biofilm formation.

### 3.5 Bacterial functions in single and multi-species biofilms

Metatranscriptomics was performed to elucidate the mechanisms underlying the significant increase in two-species biofilm biomass. PCoA of normalized RNA counts revealed distinct gene expression profiles in the two-species biofilm (Fig. S9a), with 7595 genes upregulated compared to *A. deltaense* LSQ16 (Fig. S9b) and 6473 genes upregulated relative to *B. velezensis* WB (Fig. S9c).

**Fig. 5:**
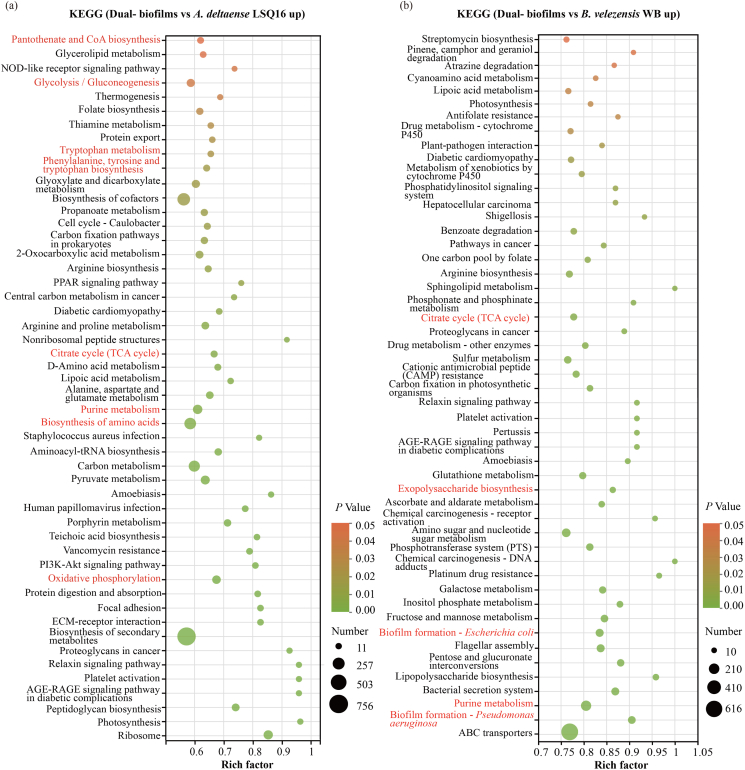
KEGG functional enrichment analysis of differentially expressed genes (DEGs). (a-b) KEGG enrichment analysis of DEGs upregulated in co-culture compared to those in *A. deltaense* LSQ16 and *B. velezensis* WB monocultures. Red labels in the figure indicate pathways of particular interest, including those related to biofilm formation, EPS biosynthesis, and amino acid metabolism. (For interpretation of the references to colour in this figure legend, the reader is referred to the Web version of this article.)

KEGG enrichment analysis revealed that, compared with *A. deltaense* LSQ16 monoculture, the DEGs in co-culture were significantly enriched in pathways including pantothenate and CoA biosynthesis, glycolysis/gluconeogenesis, tryptophan metabolism, phenylalanine, tyrosine and tryptophan biosynthesis, and oxidative phosphorylation (Fig. 5a). Relative to *B. velezensis* WB monoculture, enriched pathways included biofilm formation, extracellular polymer biosynthesis, the TCA cycle, and plant-pathogen interaction (Fig. 5b). GO functional annotation revealed that the DEGs were mostly enriched in pathways related to amino acid metabolic processes, ATPase activity, cellular metabolism, and transmembrane transport (Fig. S10a–b).

**Fig. 6:**
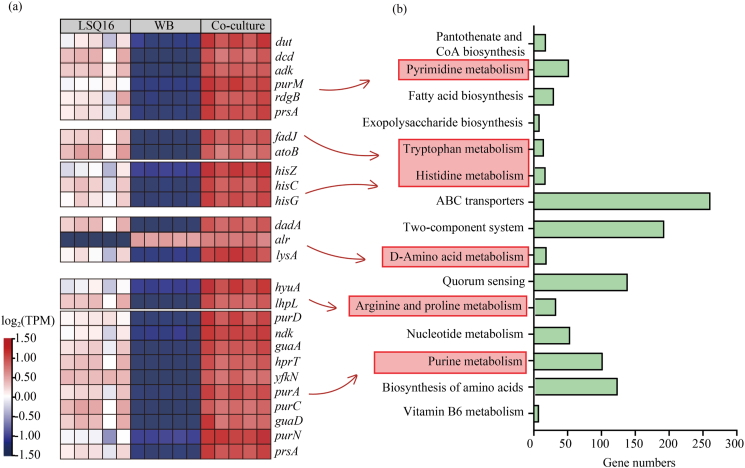
Metatranscriptomic analysis of differentially expressed genes (DEGs) under co-culture conditions for the two strains. (a) Overview of differentially expressed genes between monoculture and co-culture for the two strains. (b) KEGG pathway analysis of differentially expressed genes under co-culture and monoculture conditions for the two strains.

Further analysis was conducted on the gene expression of pathways related to amino acid, purine, and pyrimidine metabolism. Compared with monocultures, the genes involved in purine (*purD*, *ndk*, *guaA*) and pyrimidine (*dut*, *dcd*, *adk*) metabolism were upregulated in the co-culture. Genes associated with histidine metabolism (*hisZ*, *hisC*, *hisG*), d-amino acid metabolism (*dadA*, *alr*, *lysA*), and arginine and proline metabolism (*hyuA*, *lhpL*) also exhibited significantly higher relative expression levels in the co-culture (Fig. 6). These alterations in gene expression were consistent with the metabolomic results, indicating that cellular metabolic activity had undergone significant alterations under co-culture conditions.

**Fig. 7:**
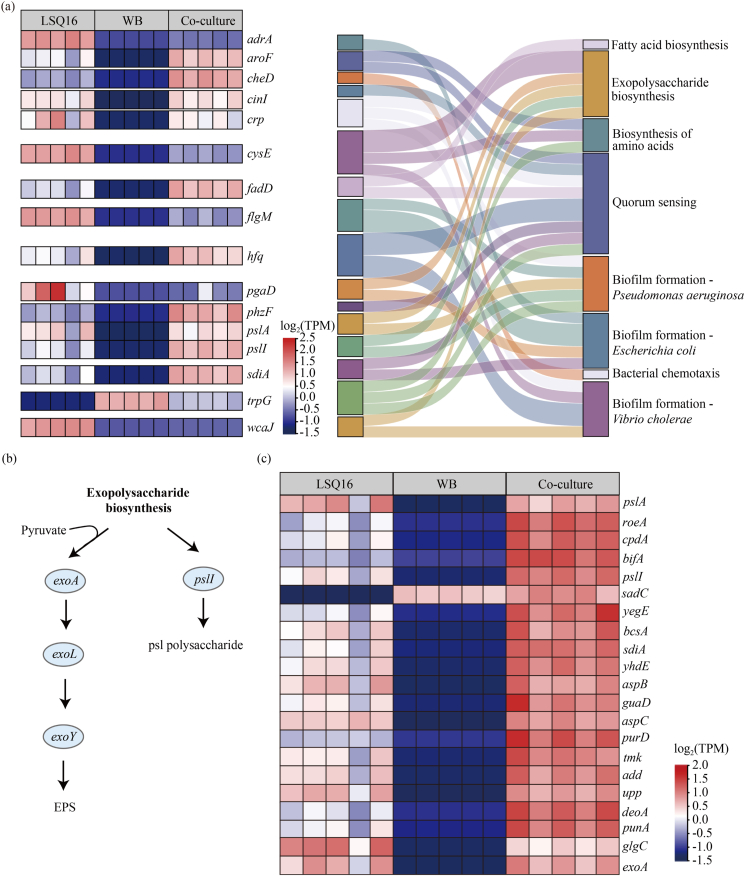
Overview of differentially expressed genes in monoculture and co-culture. (a) Heatmap analysis of up- and downregulated genes in co-culture versus monoculture (left) and the KEGG pathways associated with these genes (right). (b) Changes of genes involved in exopolysaccharide biosynthesis in co-culture. (c) Heatmap analysis of genes related to biofilm formation in co-culture.

Compared with monocultures, co-culturing *A. deltaense* LSQ16 and *B. velezensis* WB significantly increased the expression of Psl biosynthetic genes *pslA* and *pslI* (Fig. 7a). Expression of EPS synthesis-related genes, such as *exoA*, was also elevated under co-culture conditions. Nucleotide metabolism genes (*aspB*, *guaD*, *aspC*, *purD*, *tmk*, *add*, *upp*, *deoA*, *punA*) were also upregulated in co-culture (Fig. 7b–c). The expression of multiple biofilm formation-related genes, including *pslA*, *roeA*, *cpdA*, *bifA*, *pslI*, *sadC*, *yegE*, *bcsA*, *sdiA*, and *yhdE*, was also significantly upregulated (Fig. 7c). The synergy between the two strains enhanced gene expression, which promoted biofilm formation and stability.

### 3.6 Supplementation of key exchanged metabolites revealed physiological benefits of cross-feeding

**Fig. 8:**
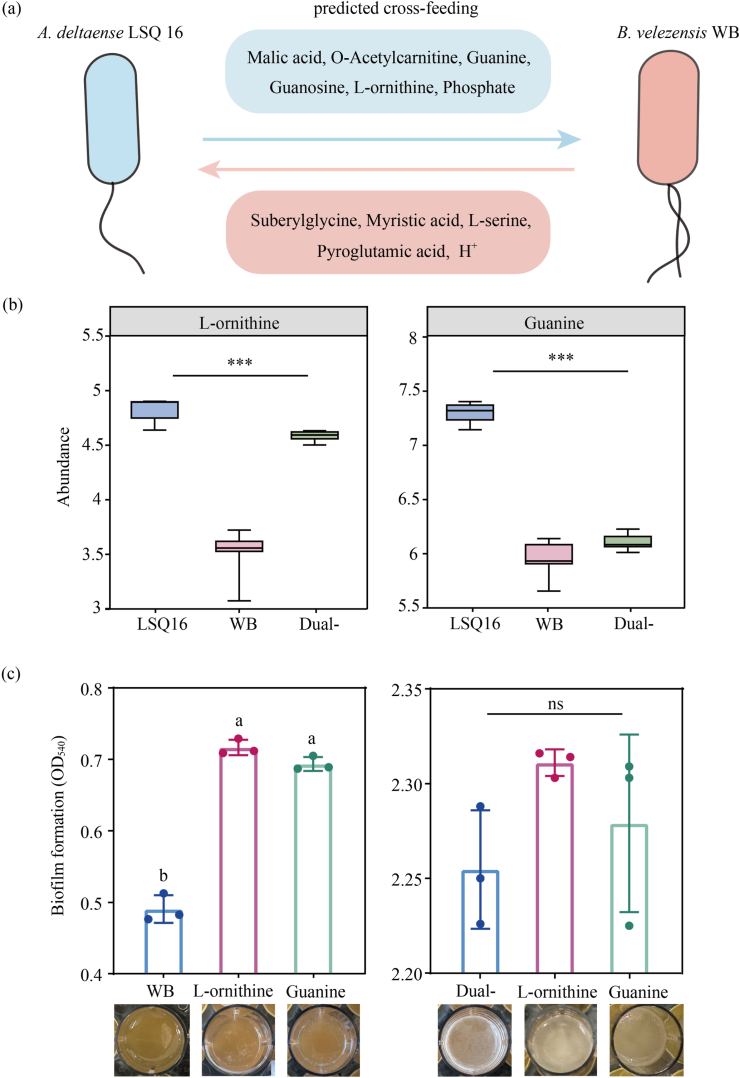
GEMs prediction and validation. (a) Exchange metabolites between two strains based on GEMs prediction. (b) Comparison of l-ornithine and guanine content between co-culture and monoculture. Statistical significance is indicated by asterisks. ∗∗∗*P* < 0.001. (c) *B. velezensis* WB and SynCom Y biofilm biomass after supplementation with l-ornithine and guanine. Statistical testing was performed using a one-way ANOVA with Tukey's multiple comparisons test (*P* < 0.05). Data represents the mean ± SD of n = 3 biological replicates. “ns" indicates no significant difference.

Genome-scale metabolic models were reconstructed to predict potential exchanged metabolites between the two species (Dataset S1). *A. deltaense* LSQ16 may supply malate, O-acetylcarnitine, guanine, guanosine, l-ornithine, and phosphate to *B. velezensis* WB, which in turn provides suberoylglycine, myristic acid, l-serine, and pyroglutamic acid to *A. deltaense* LSQ16 (Fig. 8a). Comparison with metabolomic data showed that, in co-culture, l-ornithine and guanine levels were higher than those in *B. velezensis* WB but lower than those in *A. deltaense* LSQ16 monocultures (Fig. 8b). In contrast, the levels of l-serine and pyroglutamic acid levels were higher than in *A. deltaense* LSQ16 but lower than those in *B. velezensis* WB (Fig. S11). These results suggest a bidirectional and mutually beneficial nutritional interaction between the two strains. The supplementation of l-ornithine and guanine led to an increase in the biofilm biomass of *B. velezensis* WB (Fig. 8c). In contrast, the addition of l-serine, pyroglutamic acid, and other compounds did not result in a significant enhancement of the biofilm biomass in *A. deltaense* LSQ16 monocultures (Fig. S12). These results indicate that *A. deltaense* LSQ16 promotes biofilm formation by exchanging metabolites with *B. velezensis* WB.

## 4 Discussion

In natural environments, microorganisms are frequently exposed to diverse stresses. Numerous studies have demonstrated that synthetic microbial communities are more effective than single strains in enhancing plant stress tolerance [16,35,36]. Bacteria enhance their resistance to adverse environmental conditions through synergistic mechanisms such as cross-feeding [37], chemical signaling [38], and the formation of biofilms [39,40], which serve as protective communal structures [41]. These strains interact to form complex multispecies biofilms on the rhizosphere or soil surface [42], which can protect plants by covering leaves or colonizing the root zone to prevent pathogen invasion [43]. Such biofilms play a crucial role in regulating the soil microenvironment and maintaining plant health. Using a stepwise strain-omission strategy, this study ultimately identified a two-strain consortium composed of *A. deltaense* LSQ16 and *B. velezensis* WB that exhibited enhanced biofilm biomass. We validated the key metabolites exchanged between the two strains, demonstrating a nutritional relationship between *A. deltaense* LSQ16 and *B. velezensis* WB. Meanwhile, *A. deltaense* LSQ16 can act as a partner of *B. velezensis* WB to reduce the incidence of plant diseases. Such metabolic interaction patterns may be widespread in rhizosphere microbial communities. For example, *B. velezensis* SQR9 enriches biocontrol-associated *Lysobacter* spp., and their co-culture promotes synergistic biofilm formation and antifungal metabolite production via cross-feeding, enhancing Fusarium wilt suppression [44]. The dual-species consortium composed of *Pseudomonas protegens* Δ*pltB* and *Bacillus velezensis* DMW1 also enhanced biofilm formation through synergistic interactions, thereby more effectively controlling tomato bacterial wilt [19]. These results further highlight the importance of microbial interactions in biological control.

Interactions among bacteria play a crucial role in shaping microbial community functions [45]. Moreover, conducting a systematic investigation of these interspecies interactions is of great significance [46]. This study investigated antagonistic interactions between strains from two perspectives: biofilm formation at the air–solid interface and cross-feeding of metabolites. The results indicated that *B. velezensis* LSQ19 exhibits competitive interactions with multiple strains (Appendix A, Appendix Aa–b). Meanwhile, synergistic interactions were observed between *A. deltaense* LSQ16 and *B. velezensis* WB, *E. ludwigii* LSQ1, and *A. pittii* LSQ3 (Fig. 1a–b), allowing mutual promotion of growth. Therefore, the SynCom Y, composed of *A. deltaense* LSQ16 and *B. velezensis* WB, was selected for subsequent studies. Co-cultivation of the strains significantly increased biofilm biomass, which steadily grew over time and reached its maximum within 72 h (Fig. 1d–g). Quantitative PCR under co-culture conditions confirmed that mixed-species biofilm formation promotes the growth of both strains (Fig. 2a–b), indicating interspecies interactions between them. Bacterial cell-free supernatants contain secreted nutrients, including amino acids and sugars, which function as chemoattractants [47]. These compounds direct surrounding bacteria toward regions of higher nutrient concentrations, thereby enhancing their chemotactic behavior. *B. velezensis* WB exhibited a pronounced chemotactic interaction toward *A. deltaense* LSQ16 (Fig. 2c–d). This was further supported by the upregulation of chemotaxis-related genes, such as *cheD*, under co-culture conditions (Fig. 7a). Compared with single-species biofilms, the formation of multi-species biofilms can enhance bacterial functionality and environmental adaptability [48,49]. The dual-strain combination secreted more EPS (Fig. 2e), which corresponds to a stronger capacity for biofilm formation. Meanwhile, the growth and proliferation of microbial communities within biofilms, together with increased extracellular polymer secretion, typically lead to enhanced metabolic activity [50]. Under co-culture conditions, metabolic activity was significantly increased (Fig. 2f), suggesting that the consortium has a robust metabolic capacity.

Plant roots secrete a variety of compounds, including organic acids, amino acids, sugars, and other small molecules, along with macromolecules such as proteins and polysaccharides [51]. These exudates strongly attract soil microorganisms and have a pronounced impact on bacterial chemotaxis and biofilm formation. The addition of root exudates during the cultivation of mixed-species biofilms was found to supply nutrients, thereby promoting the biofilm-forming ability of the consortium (Fig. 3a–b). However, in vitro conditions differ from the plant rhizosphere. Whether the two strains form a synergistic relationship in the rhizosphere still requires validation via root colonization assays. Future work will focus on this to elucidate the interaction mechanisms of SynCom Y in the rhizosphere. When the biocontrol bacteria encounter the pathogen *Fusarium oxysporum* f. sp. *niveum* (*Fon*), the pathogen secretes fusaric acid (FA) to resist the biocontrol strains. The accumulation of EPS forms a physical barrier, which aids in maintaining a stable microenvironment around the cells [52,53]. When subjected to FA stress, dual-species biofilms demonstrated greater resistance than single-species biofilms (Fig. 3e). Although this study did not perform LC-MS identification of FA actually secreted by *Fusarium oxysporum* f. sp. *niveum* during watermelon infection, previous studies have confirmed that in *Fusarium*-related pathogenic systems, the secreted FA matches the chemical structure of the Sigma-Aldrich FA standard [54]. In host-pathogen interaction systems, the production of metabolites and their interactions with fungal or plant-derived compounds may still differ. To validate the findings under conditions closer to natural environments, we further conducted pot experiments. SynCom Y significantly reduced disease incidence, showing superior biocontrol compared with the control (Fig. 3d and f, Table S4). This indicates that SynCom Y possesses higher environmental adaptability and stability through synergistic interaction of two strains, thereby reducing the incidence of Fusarium wilt.

Bacterial interactions, including cooperation and competition, within mixed communities can drive changes in metabolites and gene expression [28]. Some strains may rely on specific metabolites provided by other strains, such as amino acids [16,55], vitamins [22,56], or antibiotics, to sustain growth and biofilm formation. Co-culturing markedly influences the metabolite profiles within biofilms (Fig. 4a). Amino acid exchange within the consortium promotes synergy [57], and nutrients from coexisting strains enhance community survival [16]. In co-culture, higher levels of amino acids, as well as purine and pyrimidine metabolites, may also support biofilm formation [9]. Active amino acid metabolism in mixed-species biofilms reflects efficient resource utilization via metabolic complementation, further enhancing biofilm environmental adaptability and stability [58]. Certain secondary metabolites, such as neomycin, were significantly enriched in mixed-species biofilms (Fig. 4), suggesting that microbes within the mixed-species biofilms work together to produce more antimicrobial or other functional metabolites. This likely enhances resistance against pathogens, thereby improving biocontrol efficacy. Enrichment of quorum sensing (QS) and two-component system pathways in mixed-species biofilms suggests that microbial signaling is key to biofilm formation and function (Fig. 4e). These signaling pathways optimize biofilm structure and function by regulating gene expression and metabolic pathways, thereby enhancing the biofilms’ resistance to external stresses.

Metatranscriptomic analysis revealed significant differences in gene expression between single-species and mixed-species biofilms, with differentially expressed genes (DEGs) in mixed-species biofilms primarily associated with purine metabolism, pyrimidine metabolism, and amino acid biosynthesis (Fig. 5, Fig. 6). This suggests that co-culture conditions increase cellular metabolic demands to accommodate the symbiotic relationship [59]. Significant differences were observed in the expression of genes related to EPS synthesis and biofilm formation between monocultures and co-cultures. In mixed-species biofilms, the synergistic interactions among multiple microbes enhance biofilm formation and functionality by regulating the expression of specific genes [9,60]. Psl polysaccharide is a major component of the biofilm matrix [61]. Genes related to EPS synthesis, such as *pslA*, *pslI*, and *exoA*, were significantly upregulated in mixed-species biofilms (Fig. 7b), indicating that microbial synergy enhances biofilm structural stability by promoting EPS secretion. EPS synthesis is closely associated with microbial quorum sensing and metabolic activity [62]. Changes in the expression of quorum sensing-related genes, such as *bifA*, *sadC*, and *aspB*, also indicate that microbes in mixed-species biofilms can coordinate their behaviors more effectively, thereby optimizing biofilm functionality (Fig. 7c). Genes associated with the regulation of the second messenger c-di-GMP (such as *sdiA* and *yegE*) were also significantly upregulated in mixed-species biofilms. c-di-GMP drives biofilm formation by increasing the production of extracellular polysaccharides and adhesins while simultaneously downregulating flagella-based motility [63]. These gene expression changes suggest that microbes in mixed-species biofilms enhance biofilm formation by cooperatively regulating c-di-GMP levels and extracellular matrix synthesis.

Nutrient sources such as carbon and nitrogen influence bacterial growth rates and the levels of proteins and polysaccharides in the biofilm matrix, thereby affecting biofilm formation [64]. Potential metabolite exchanges between *A. deltaense* LSQ16 and *B. velezensis* WB were predicted using GEMs (Fig. 8a, Dataset S1). To further identify key metabolites, those showing significant abundance changes in the metabolomic data were tested as additional carbon sources in vitro. The results indicate that *A. deltaense* LSQ16 supplies l-ornithine and guanine to *B. velezensis* WB, significantly enhancing its biofilm biomass (Fig. 8b–c). At the application level, *Bacillus* spp. have been widely developed as biofertilizers and microbial inoculants, primarily promoting plant growth and enhancing disease resistance through rhizosphere colonization and the production of bioactive metabolites. This study shows that l-ornithine and guanine enhance biofilm formation in *B. velezensis* WB, suggesting their potential as biofertilizer enhancers by promoting rhizosphere colonization and functionality. Future work should validate these effects under field conditions. Overall, these findings highlight the importance of microbial cooperation in promoting biofilm formation and improving disease control.

## 5 Conclusion

This study identified a synergistic consortium of *A. deltaense* LSQ16 and *B. velezensis* WB with enhanced biofilm-forming ability in vitro. Under co-culture conditions, SynCom Y exhibited increased EPS secretion, enhanced metabolic activity, and improved resistance to FA as well as to the pathogen causing Fusarium wilt in watermelon. Enhanced biofilm formation in SynCom Y is primarily linked to metabolic pathways such as amino acid and purine and pyrimidine metabolism, with co-culture upregulating the expression of related genes. Metabolic exchange occurs between the two strains, and genome-scale predictions of metabolite exchange were validated in vitro. Exogenous l-ornithine and guanine supplementation significantly enhanced *B. velezensis* WB biofilm formation in vitro. These findings indicate that the functional microbial consortium enhances biofilm biomass through synergistic interactions, providing a theoretical basis for biofilm-based biocontrol approaches.

## CRediT authorship contribution statement

**Yue Zhang:** Writing – original draft, Visualization, Validation, Software, Data curation. **Zhigang Wang:** Methodology, Investigation, Conceptualization. **Weihui Xu:** Writing – review & editing, Supervision, Project administration, Funding acquisition, Conceptualization.

## Funding

The authors gratefully acknowledge the funding of this work by the Projects of Key Research and Development Plans in Heilongjiang Province, China (GA23B018) and the 10.13039/501100002367Chinese Academy of Sciences.

## Declaration of competing interest

The authors declare the following financial interests/personal relationships which may be considered as potential competing interests: Xu Weihui reports financial support was provided by The Projects of Key Research and Development Plans in Heilongjiang Province, China (GA23B018) and the Chinese Academy of Sciences. Reports a relationship with that includes:. Has patent pending to. If there are other authors, they declare that they have no known competing financial interests or personal relationships that could have appeared to influence the work reported in this paper.

## Data Availability

Data will be made available on request.
