# Supplementary material for: Biofilm synergy by Agrobacterium deltaense and Bacillus velezensis in co-cultures indicates bacterial interspecific cooperation
Source: Biofilm. 2026 May 4;11:100364. doi: 10.1016/j.bioflm.2026.100364 (PMC13158395; doi:10.1016/j.bioflm.2026.100364)
Supplement: Multimedia component 1 [file mmc1.doc]

**Supplementary material captions**

**LC-MS non-targeted metabolomics instrument parameter settings.**

A 2 μL sample was separated using an HSS T3 column (100 mm × 2.1 mm i.d., 1.8 µm) and subsequently analyzed by mass spectrometry. Mobile phase A consists of 95% water and 5% acetonitrile (containing 0.1% formic acid), while mobile phase B comprises 47.5% acetonitrile, 47.5% isopropanol, and 5% water (also containing 0.1% formic acid). Separation gradient: From 0 to 0.1 minutes, mobile phase B is linearly increased from 0% to 5%; from 0.1 to 2 minutes, mobile phase B is linearly increased from 5% to 25%; from 2 to 9 minutes, mobile phase B is linearly increased from 25% to 100%; from 9 to 13 minutes, mobile phase B is maintained at 100%; from 13.0 to 13.1 minutes, mobile phase B is linearly decreased from 100% to 0%; and from 13.1 to 16 minutes, mobile phase B is maintained at 0%. The flow rate is set at 0.40 mL/min, with the column temperature maintained at 40°C. Mass spectrometric signal acquisition for the samples was performed in both positive and negative ion scanning modes, with a mass scanning range of m/z 70-1050. The ion spray voltage was set to 3500 V for positive ions and 2800 V for negative ions. The sheath gas pressure was maintained at 40 psi, with auxiliary heating gas at 10 psi. The ion source heating temperature was set to 400°C. The collision energies were cycled at 20-40-60 V, with an MS1 resolution of 70,000 and an MS2 resolution of 17,500.

**Table S1** Identification of six soil isolates by 16S rRNA analysis.

| Strain no. | Culturomics-matched Species/Genus | 16S rRNA identification |
| --- | --- | --- |
| LSQ1 | *Devosia* | *Enterobacter ludwigii* |
| LSQ3 | *Niabella* | *Acinetobacter pittii* |
| LSQ14 | *Chitinophaga* | *Chitinophaga silvisoli* |
| LSQ16 | *Pelagibacterium* | *Agrobacterium deltaense* |
| LSQ19 | *Devosia* | *Bacillus velezensis* |
| WB | — | *Bacillus velezensis* |

**Table S2** Test medium.

| Name | Ingredients |
| --- | --- |
| TSB medium | Tryptone 17.0 g, Soy peptone 3.0 g, NaCl 5.0 g,  K_2_HPO_4_ 2.5 g, Glucose 2.5 g |
| LBGM medium | Peptone 10.0 g, NaCl 8.0 g, Yeast paste 5.0 g, MnSO_4_ 0.1 mmol/L, 1% Glycerol, add water to make 1000 mL (pH=7.0) |

**Table S3** The specific primer set of *A. deltaense* LSQ16 and *B. velezensis* WB.

| Strain | Primers | Sequence (5’-3’) |
| --- | --- | --- |
| LSQ16 | Forward | AAGAACGGCAACAAGGACG |
|  | Reverse | CGGTAATAATCTGCAAGGGAAA |
| WB | Forward | CAGGACAACGGCTACGACATGA |
|  | Reverse | AGAGGCGCTCCGTCACAGAAGT |

**Table S4** Effect of different treatments on the incidence of Fusarium wilt in watermelon.

| Treatment | Disease incidence (%) | Relative control efficiency (%) |
| --- | --- | --- |
| CK | 41.67±8.78 a |  |
| LSQ16 | 27.50±2.50 b | 56.62±1.87 d |
| WB | 22.50±4.33 bc | 68.42±2.10 c |
| SynCom Y | 18.33±3.81 bc | 79.13±2.47 b |
| SynCom Q | 15.83±2.89 c | 85.62±2.64 a |

Note: The different lowercase letters indicate significant differences among treatments (*P* < 0.05)


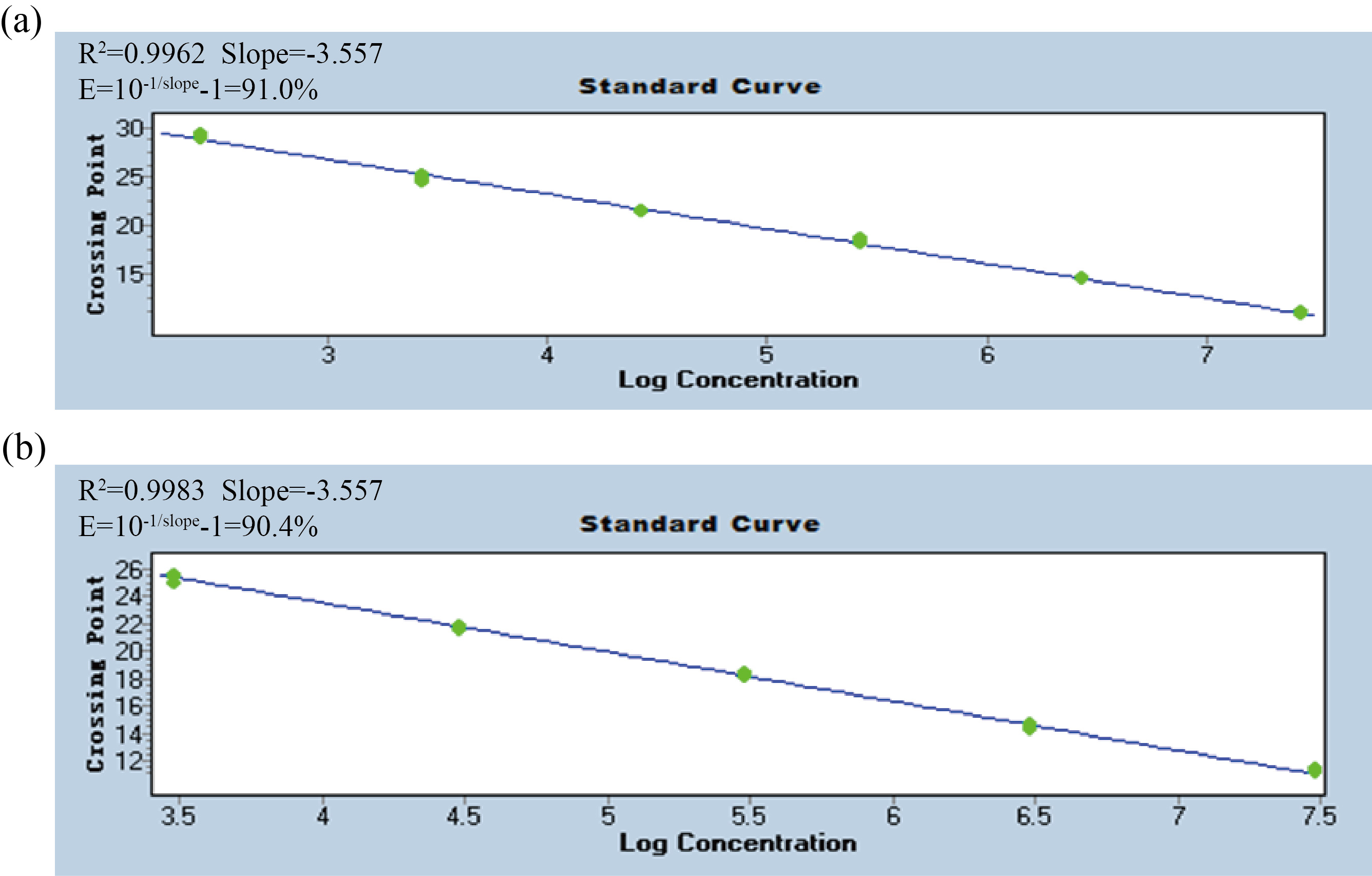


**Fig. S1** Strain-specific qPCR standard curves. (a) *A. deltaense* LSQ16; (b) *B. velezensis* WB.

**
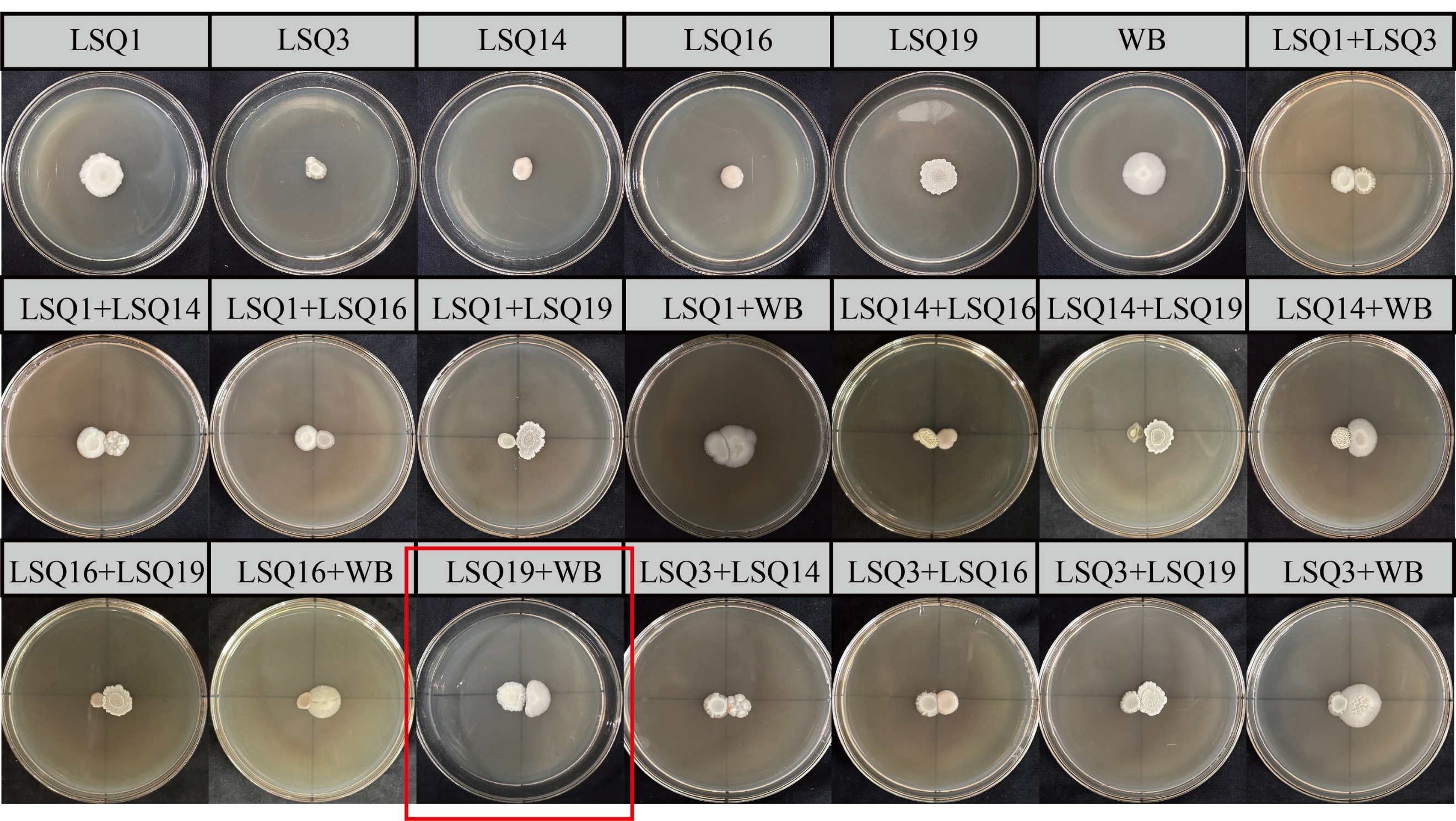
**

**Fig. S2** Biofilm formation ability of individual strains and strain combinations at the solid-gas interface over 72 h.

**
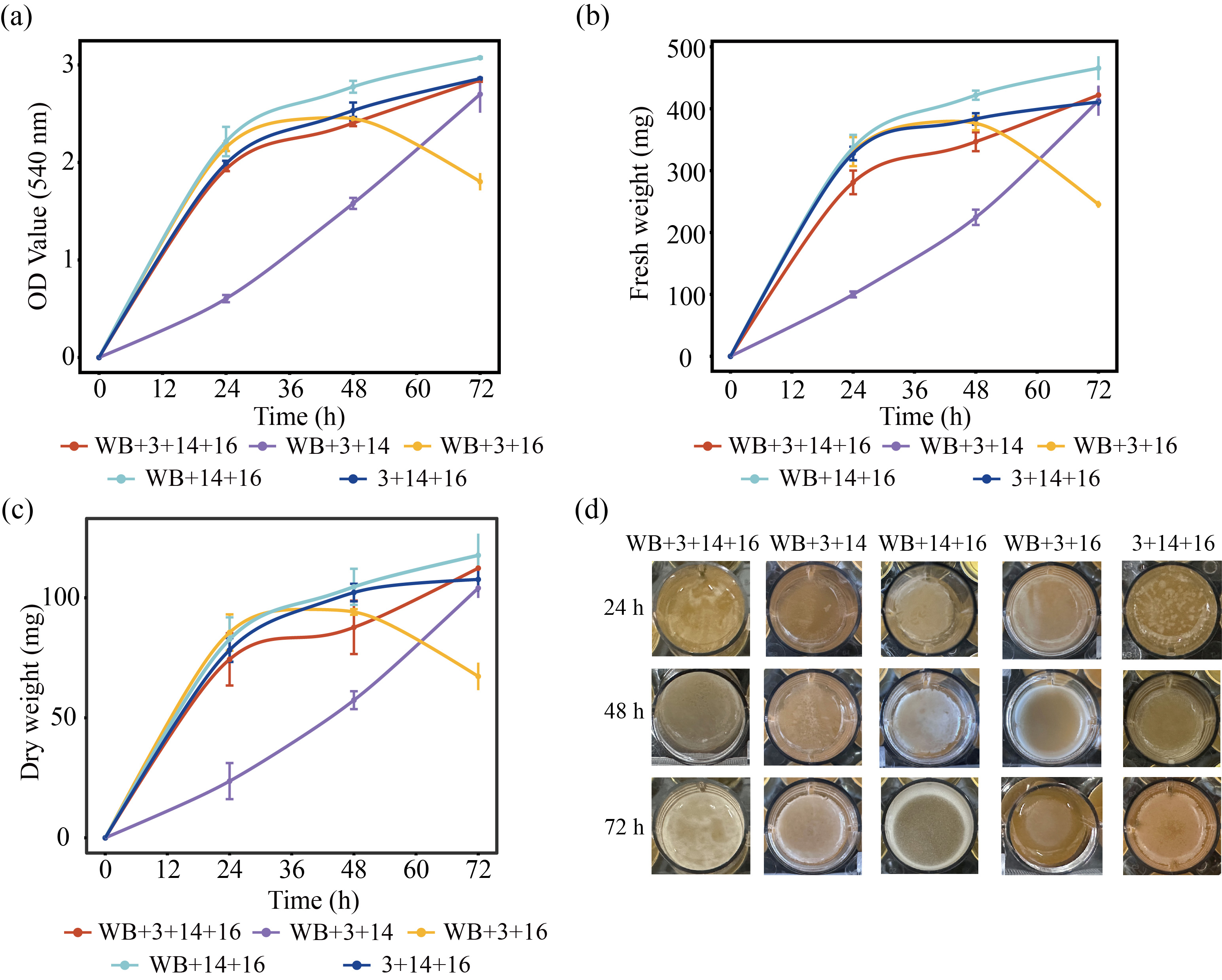
**

**Fig. S3** (a) The WB+3+14+16 consortium was sequentially simplified by stepwise strain omission. (b) Biofilm images of the WB+3+14+16 consortium following stepwise simplification through sequential strain omission.

**
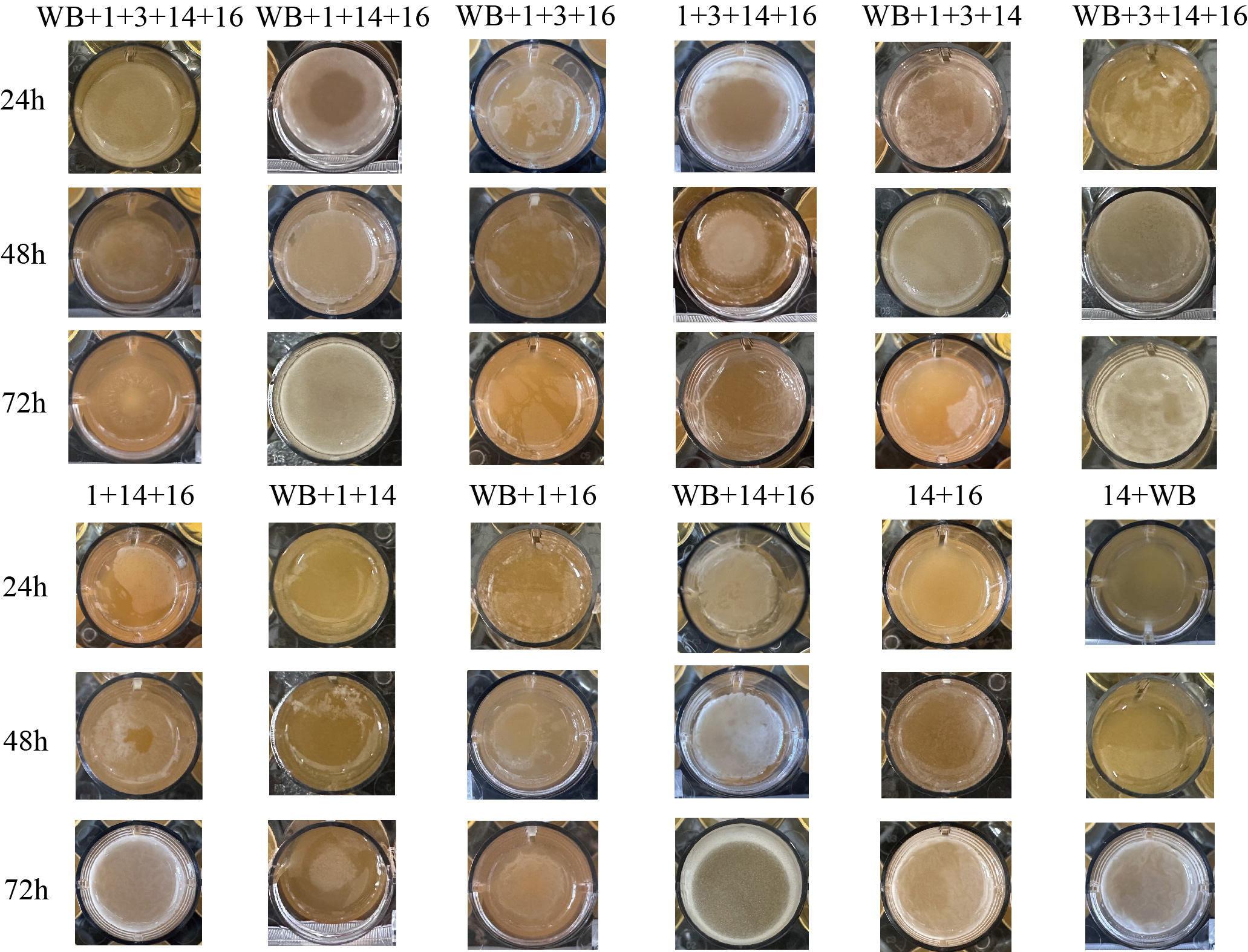
**

**Fig. S4** Biofilm images at 24 h, 48 h, and 72 h.


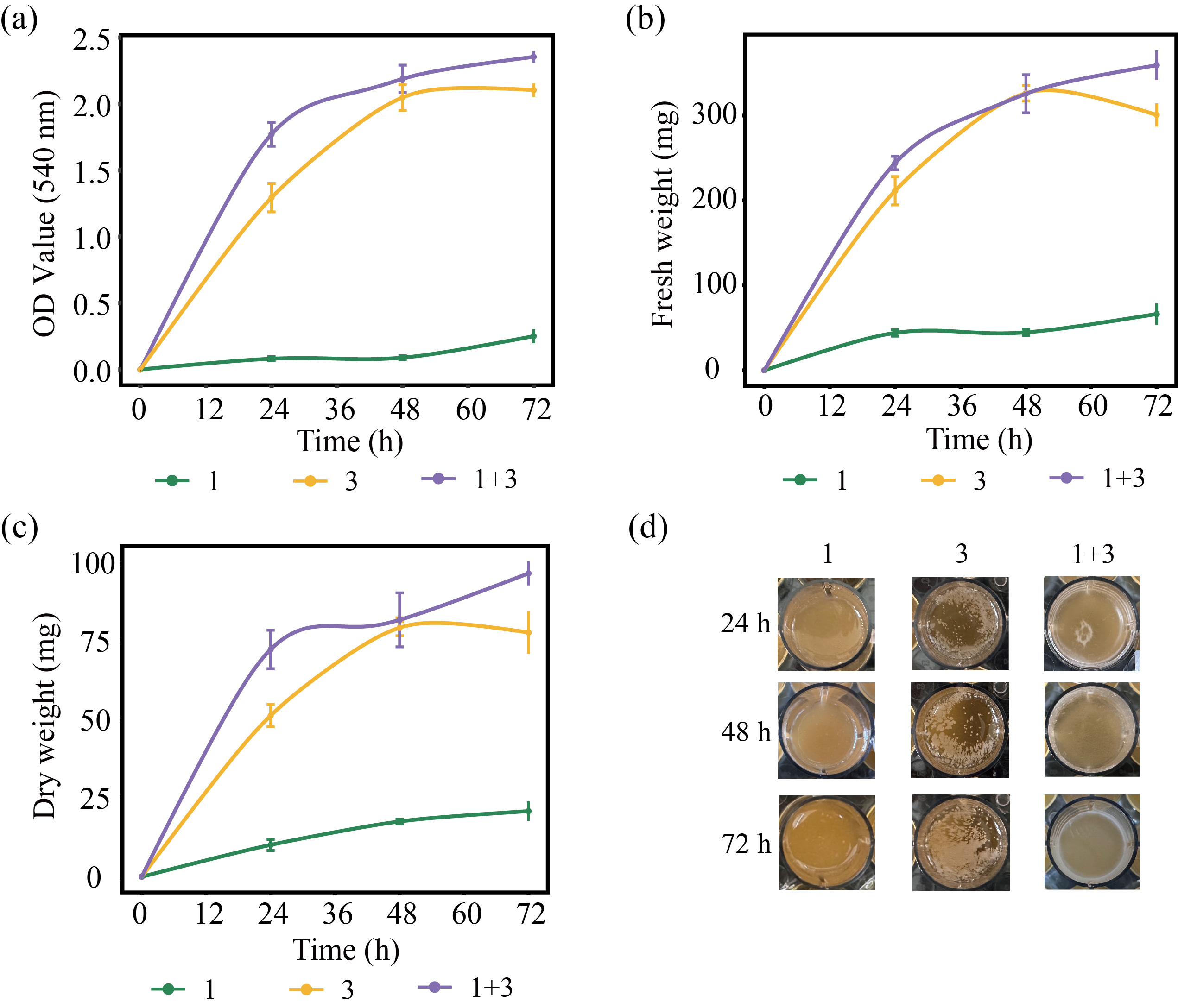


**Fig. S5** (a) Biofilm-forming capacity of strain LSQ1, strain LSQ3, and their co-culture at different time points. (b) Biofilm images of strain LSQ1, strain LSQ3, and their co-culture.


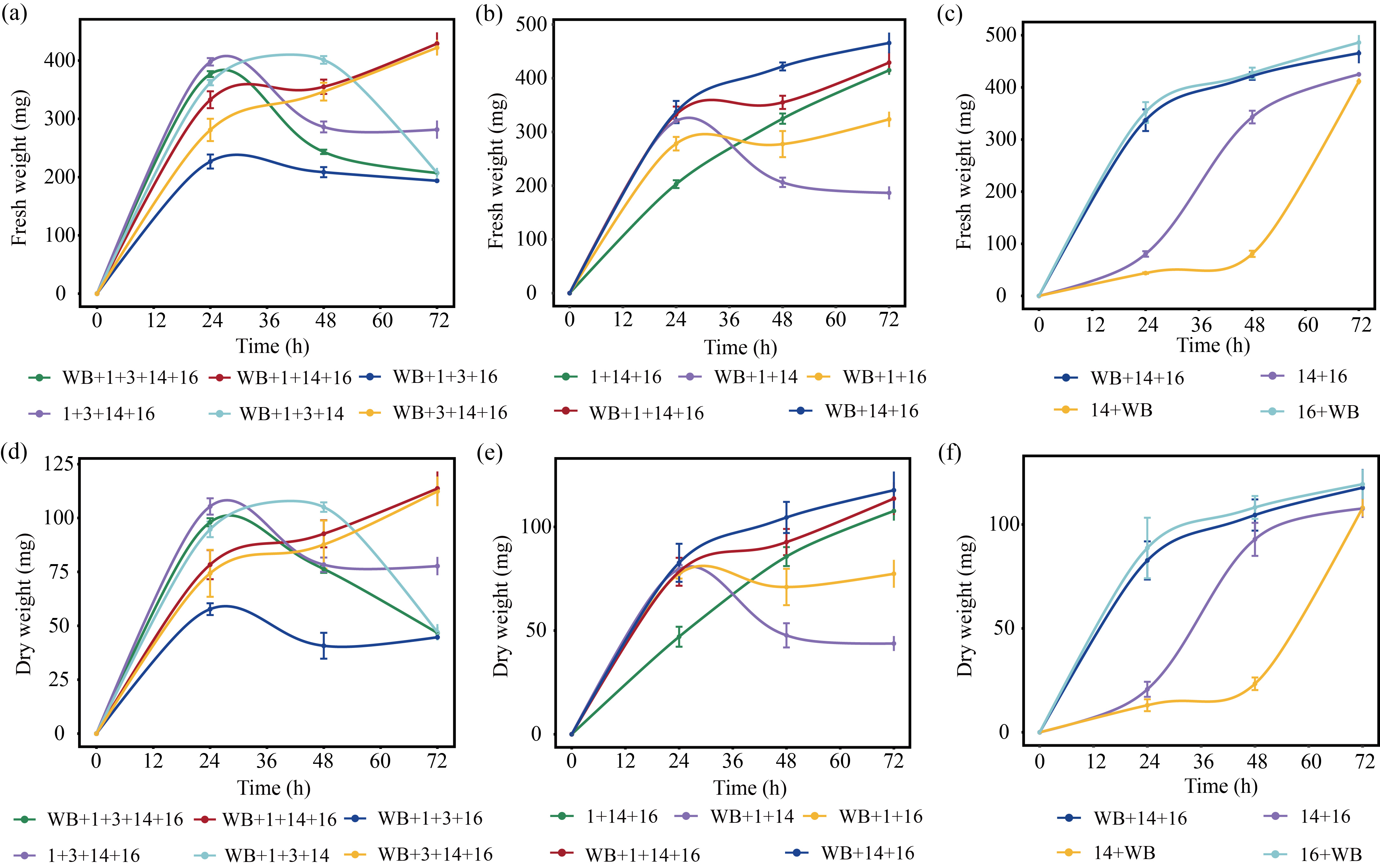


**Fig. S6** Fresh weight (a-c) and dry weight (d-f) of biofilms formed by different microbial communities at 24 h, 48 h, and 72 h.


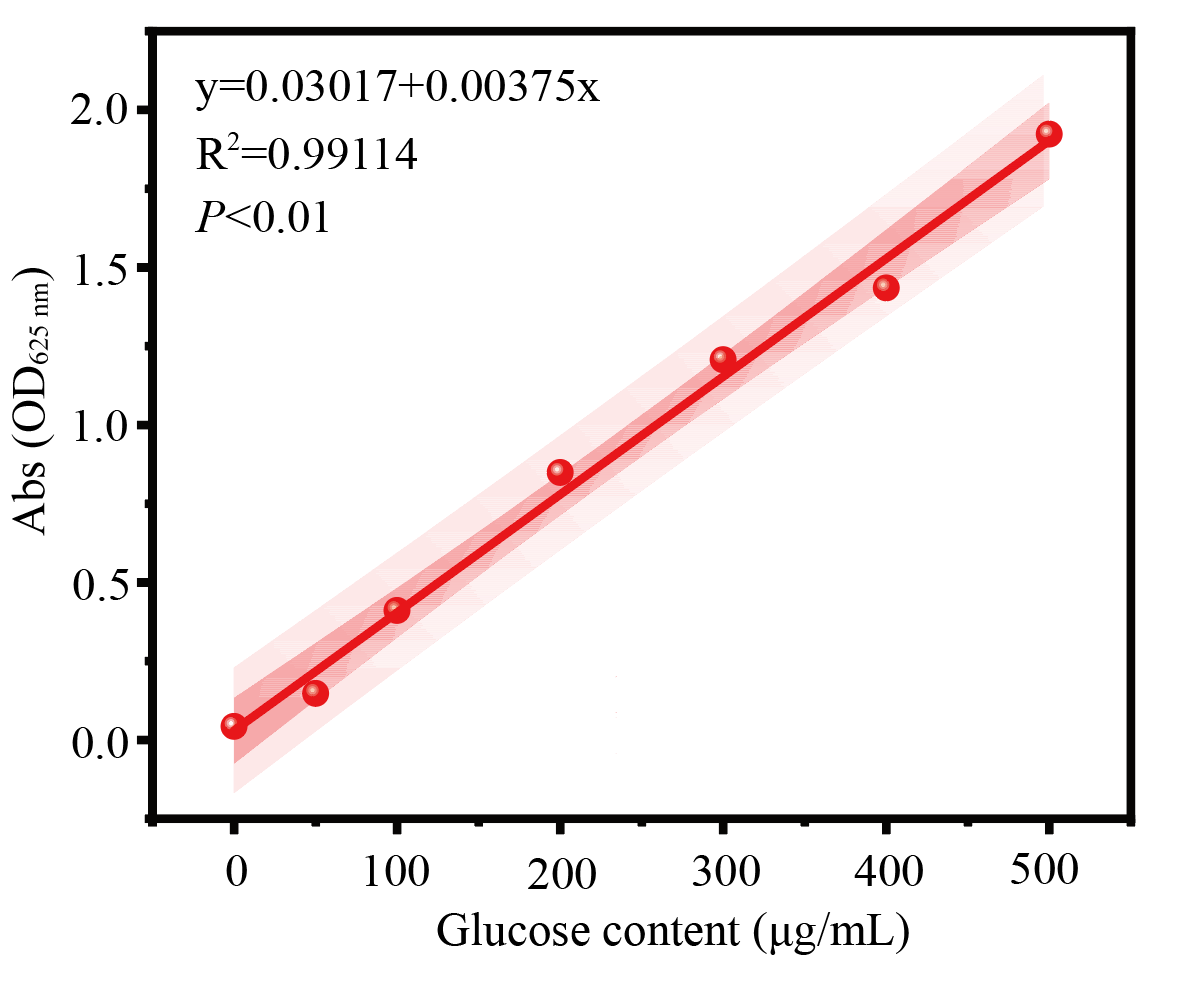


**Fig. S7** Extracellular polysaccharide standard curve.


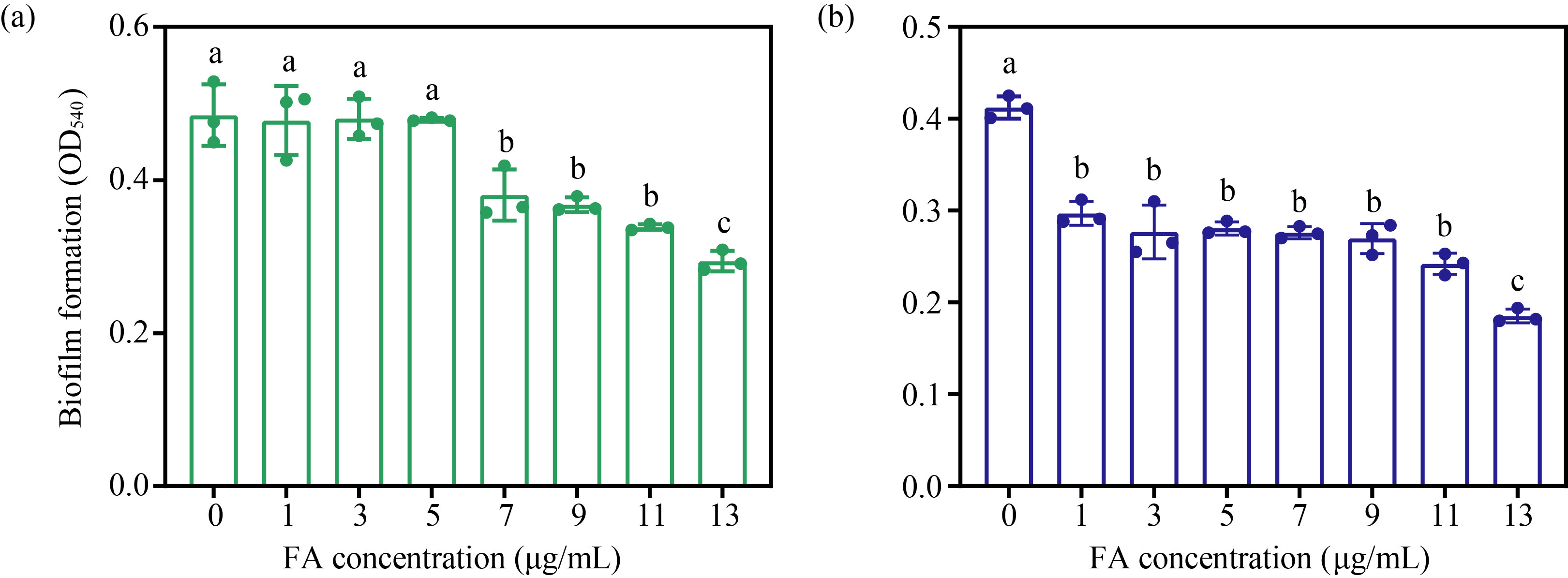


**Fig. S8** The resistance of single-species biofilms to fusaric acid (FA). (a) The resistance of *A. deltaense* LSQ16 to FA. (b) The resistance of *B. velezensis* WB to FA. Statistical analyses were performed using one-way ANOVA followed by Tukey’s multiple comparisons test (*P* < 0.05). Data represent the mean ± SD (n = 3 biological replicates).


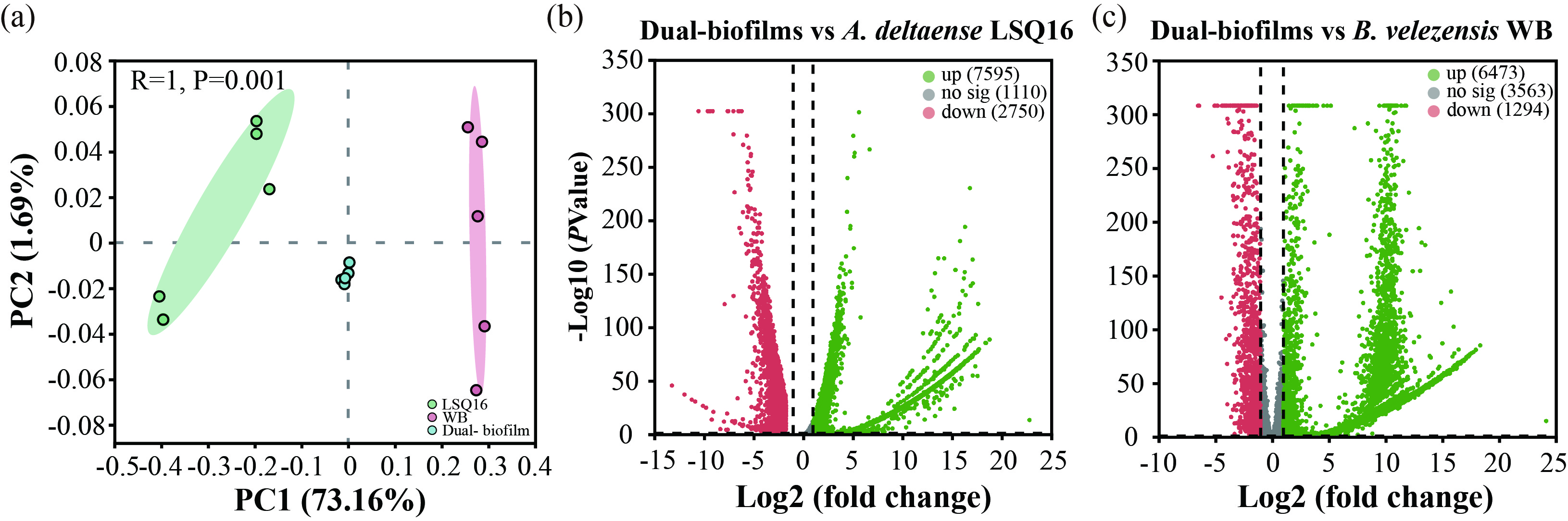


**Fig. S9** Analysis of differentially expressed genes under co-culture and monoculture conditions for the two strains. (a) PCoA analysis of single- and dual-species biofilms. (b) Volcano plot of differentially expressed genes in *A. deltaense* LSQ16 under co-culture and monoculture conditions. (c) Volcano plot of differentially expressed genes in *B. velezensis* WB under co-culture and monoculture conditions.

**
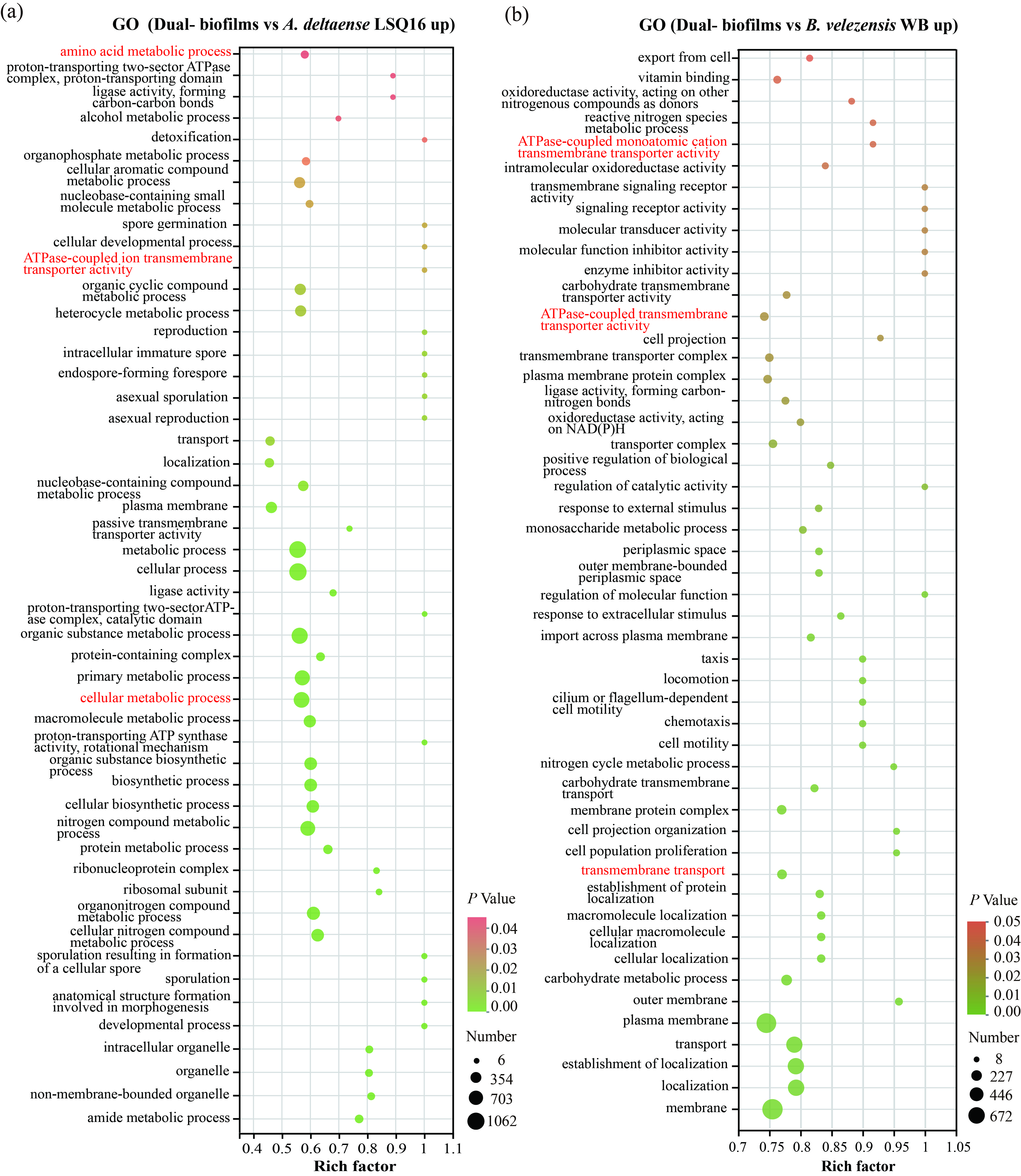
**

**Fig. S10** GO functional enrichment analysis of differentially expressed genes (DEGs). (a-b) GO enrichment analysis of DEGs upregulated in co-culture compared to those in *A. deltaense* LSQ16 and *B. velezensis* WB monocultures.

**
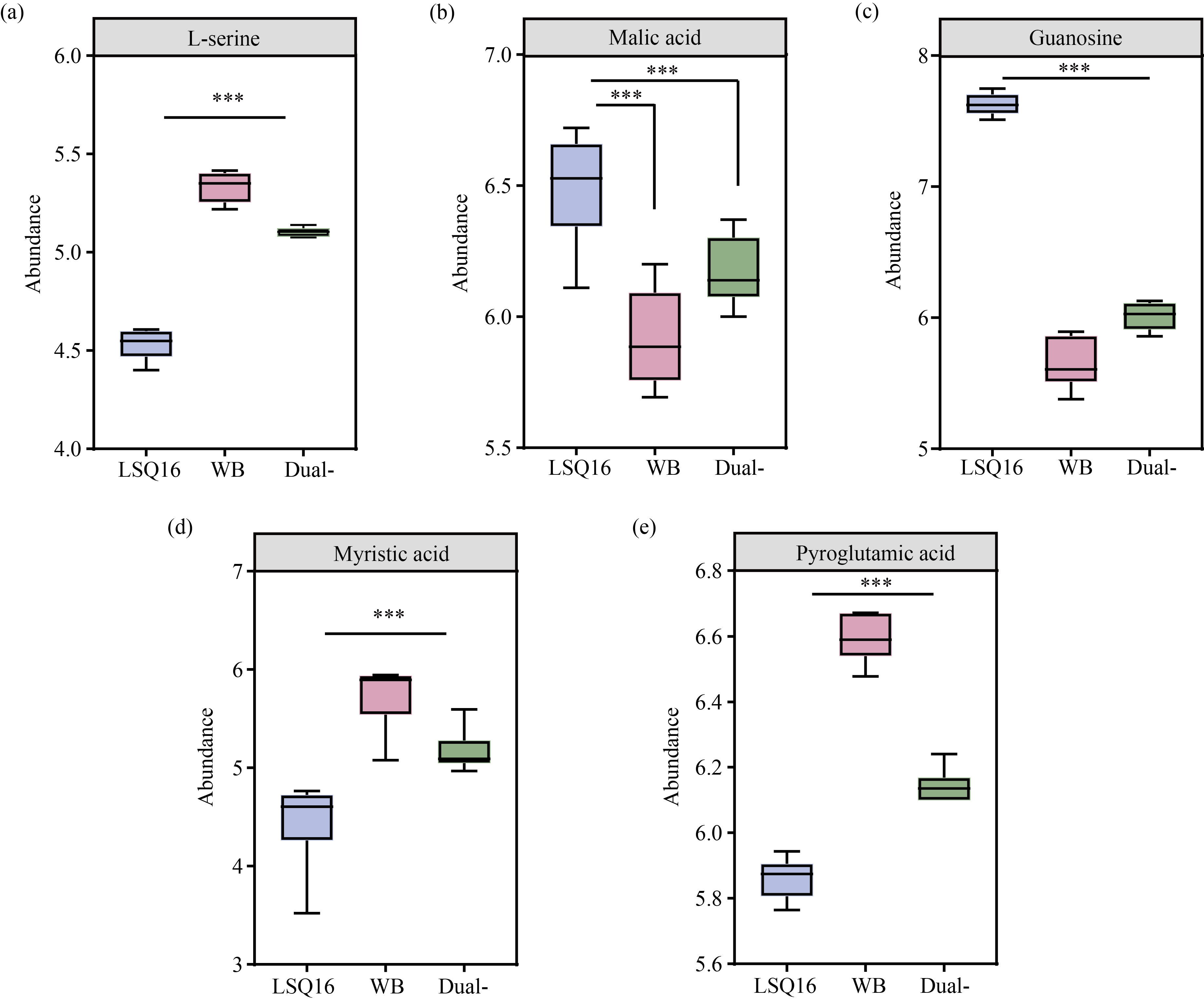
**

**Fig. S11** Comparison of metabolite abundances between co-culture and monoculture. (a) L-serine; (b) Malic acid; (c) Guanosine; (d) Myristic acid; (e) Pyroglutamic acid. Statistical significance is indicated by asterisks (****P* < 0.001).

**
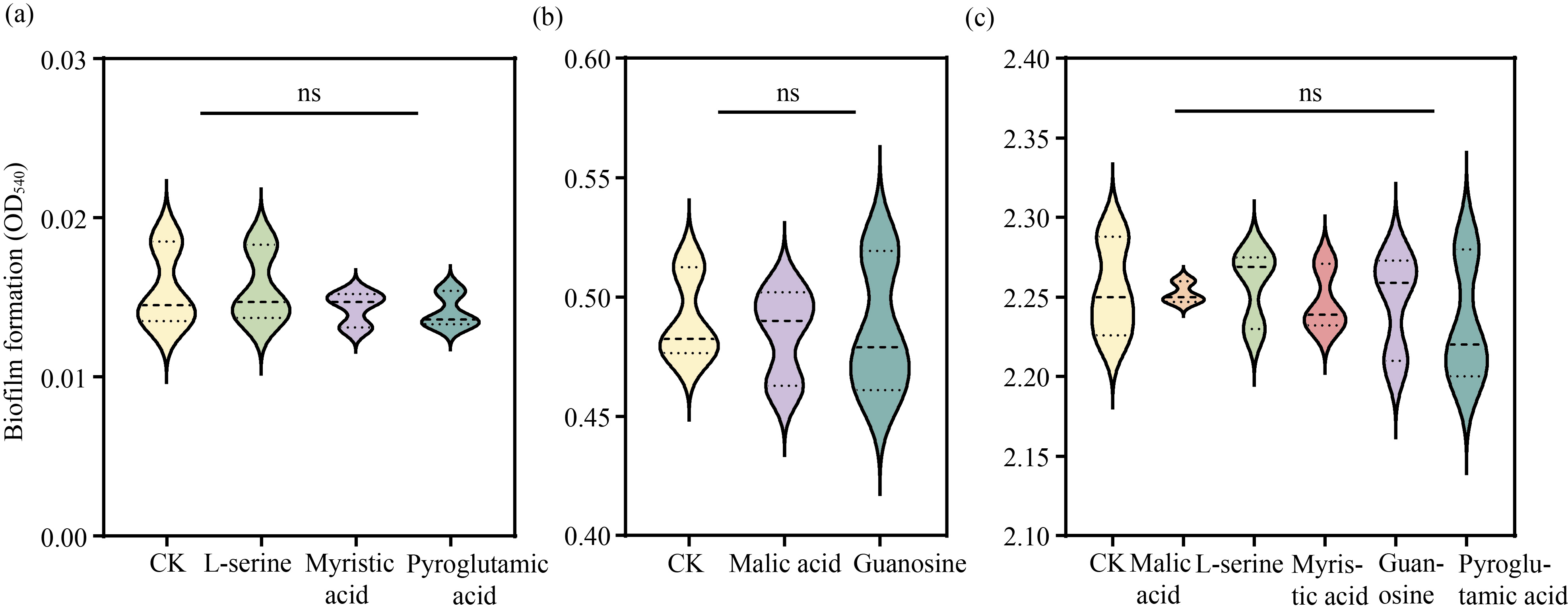
**

**Fig. S12** In vitro validation. (a) Biofilm biomass of *A. deltaense* LSQ16 after metabolite supplementation. (b) Biofilm biomass of *B. velezensis* WB after metabolite supplementation. (c) Biofilm biomass of the dual-species consortium after metabolite supplementation. Statistical analyses were performed using one-way ANOVA followed by Tukey’s multiple comparisons test. Data represent the mean ± SD (n = 3 biological replicates). “ns” indicates no significant difference.
